# Expanding the environmental virome: Infection profile in a native rainforest tree species

**DOI:** 10.3389/fmicb.2022.874319

**Published:** 2022-08-04

**Authors:** Anderson Carvalho Vieira, Ícaro Santos Lopes, Paula Luize Camargos Fonseca, Roenick Proveti Olmo, Flora Bittencourt, Letícia Maróstica de Vasconcelos, Carlos Priminho Pirovani, Fernanda Amato Gaiotto, Eric Roberto Guimarães Rocha Aguiar

**Affiliations:** ^1^Department of Biological Science, Center of Biotechnology and Genetics, Universidade Estadual de Santa Cruz, Ilhéus, Brazil; ^2^Department of Biochemistry and Immunology, Instituto de Ciências Biológicas, Universidade Federal de Minas Gerais, Belo Horizonte, Brazil; ^3^Department of Genetics, Instituto de Ciências Biológicas, Universidade Federal de Minas Gerais, Belo Horizonte, Brazil; ^4^Université de Strasbourg, CNRS UPR9022, Inserm, Strasbourg, France

**Keywords:** virome, agroforestry, metatranscriptomics, rainforest tree, *Carpotroche brasiliensis*

## Abstract

Agroforestry systems (AFS) for cocoa production combine traditional land-use practices with local biodiversity conservation, resulting in both ecological and agricultural benefits. The cacao-cabruca AFS model is widely implemented in regions of the Brazilian Atlantic Forest. *Carpotroche brasiliensis* (Raddi) A. Gray (Achariaceae) is a tree found in cabruca landscapes that is often used for reforestation and biotechnological applications. Despite its importance, we still lack information about viruses circulating in *C. brasiliensis*, particularly considering the possibility of spillover that could affect cocoa production. In our study, we analyzed the *Carpotroche brasiliensis* virome from Atlantic Forest and cacao-cabruca AFS regions using metatranscriptomics from several vegetative and reproductive organs. Our results revealed a diverse virome detecting near-complete or partial coding sequences of single- and double-stranded DNA and RNA viruses classified into at least six families (*Botourmiaviridae*, *Bromoviridae*, *Caulimoviridae*, *Genomoviridae*, *Mitoviridae*, and *Rhabdoviridae*) plus unclassified elements. We described with high confidence the near-complete and the partial genomes of two tentative novel viruses: Carpotroche-associated ilarvirus and Carpotroche-associated genomovirus, respectively. Interestingly, we also described sequences likely derived from a rhabdovirus, which could represent a novel member of the genus *Gammanucleorhabdovirus*. We observed higher viral diversity in cacao-cabruca AFS and reproductive organs of *C. brasiliensis* with preferential tropism to fruits, which could directly affect production. Altogether, our results provide data to better understand the virome in this unexplored agroecological interface, such as cacao-cabruca AFS and forest ecosystem, providing information on the aspects of virus–plant interactions.

## Introduction

The agroforestry-based management systems (agroforestry systems, AFS) aggregate types of traditional land-use practices involving the deliberate combination of crops, animals, and tree vegetation for agricultural commodities production, and are often used by small and large agricultural producers around the world (Nair, 1993, 2012). AFS usually provide the greatest agricultural gain in ecosystem services that are beneficial to productivity, and, in turn, biodiversity is maintained in the environment (Bhagwat et al., 2008; Teixeira et al., 2021). Nevertheless, plantation management must occur in areas close to the conserved forests for increased benefits (Faria et al., 2007). In Brazil, pastureland, cropland, monoculture tree plantations, and mosaics of AFS have been used in regions of younger native forests or remnants of old-growth forests to maintain the ecosystem services (Joly et al., 2014; Rosa et al., 2021).

One example of AFS use in Brazil is the cacao-AFS. Cocoa fruits are produced by the *Theobroma cacao* L. (*Malvaceae*) and are cultivated in over 606,794 hectares (ha) of Brazilian territory, responsible for producing 5% of the world’s cocoa (Gama-Rodrigues et al., 2021). Of this area, 430,051 ha are part of the Atlantic forest in the States of Bahia and Espírito Santo (Gama-Rodrigues et al., 2021). The cacao-cabruca AFS model is practiced in about 250,000 ha of Atlantic Forest in southern Bahia state, an area that holds most of the forest in northeastern Brazil (Martini et al., 2007; Sambuichi et al., 2012; Gama-Rodrigues et al., 2021). This AFS model contributes to 44% of the 259,425 tons of national cocoa produced in 2019 (Gama-Rodrigues et al., 2021). The *cabruca* system consists of random intercropping of cocoa in native forest strata, exploiting native or exotic trees to provide shade. This ecosystem environment is characterized by a high diversity of trees that may vary according to age, environmental conditions, and management of the areas (Sambuichi et al., 2009, 2012; Gama-Rodrigues et al., 2021).

*Carpotroche brasiliensis* (Raddi) A. Gray (Achariaceae) is a plant species native to the Atlantic Forest that is found in *cabruca* landscapes (Sambuichi and Haridasan, 2007). The species, whose vernacular name is *sapucainha*, is widely used in reforestation programs, environmental restoration, and agroforestry systems due to shade tolerance (Brito-Rocha et al., 2017; Cerqueira et al., 2018) and its capacity to provide resources for wildlife (Marangon et al., 2010; Zucaratto et al., 2010). The fruits of *C. brasiliensis* are consumable by wild animals, mainly rodents, and marketed to the cosmetics industry that makes use of its oil in esthetics products (Lima et al., 2020). The Chaulmoogra oil can be extracted from *C. brasiliensis* seeds, and bioactive fatty acids like chaulmoogric and hydnocarpic acids have anti-inflammatory, analgesic, and antiparasitic pharmacological properties (Sharma and Hall, 1991; Parascandola, 2003; Lima et al., 2005; dos Santos et al., 2008; Krist, 2020). Therefore, the valorization of *C. brasiliensis* is fundamental to adding economic and ecological value to the cacao-AFS, and the evaluation of aspects of the microbial biodiversity, such as the viral diversity, results in a greater understanding of these unexplored ecosystems of tree species, to avoid the reduction of native biodiversity in agroforestry systems (Alexander et al., 2014; Piasentin et al., 2014).

Advances in plant virology after the emergence of high-throughput sequencing (HTS) have accelerated the identification of novel virus species from crops and fruit trees in agricultural ecosystems, expanding the knowledge of viral epidemiology in intensive and diverse production systems such as AFS models (Maclot et al., 2020). In Brazil, due to recurrent virus outbreaks in crops of economic importance, more than 200 virus species infecting plants were cataloged and officially recognized by the International Committee on Taxonomy of Viruses (ICTV) until 2018 (Kitajima, 2020). However, little is known about the ecology of viruses infecting wild hosts in native ecosystems adjacent to the crop fields, and the latter is known as an agroecological interface (Alexander et al., 2014; Roossinck and García-Arenal, 2015; Rodríguez-Nevado et al., 2020). Moreover, there is a real risk of spillover of virus infection between plants, which is common among plant viruses and emergent species, such as cacao swollen shoot virus (CSSV) (Muller, 2016). This virus seems to have originated from indigenous forest trees that work as alternative hosts for pathogens (Posnette et al., 1950; Dzahini-Obiatey et al., 2010; Topolovec-Pintaric, 2020). Interestingly, other groups of viruses are undergoing evolutionary radiation, adapting to infect new plant species in different environments (Pagán, 2018; Moury and Desbiez, 2020).

Plant viruses may act in different ecological contexts along the evolutionary relationships established with the host. These viral interactions may lead to asymptomatic infections in latent or persistent viral cycles, through the integration of fragments derived from the viral genome into the host DNA as endogenous viral elements (EVEs) (Lefeuvre et al., 2019). EVEs may have a beneficial relationship for adaptation in environments or promote competition in plant communities by affecting virulence and host tolerance/susceptibility, and subsequently affect the epidemiological profile of viral pathology in the ecosystem (Engering et al., 2013; Lefeuvre et al., 2019; Takahashi et al., 2019). Understanding how evolutionary forces act in distinct environments, together with the anthropic influence on the emergence of diseases, can provide knowledge to forecast and avert alterations in natural ecosystems that cause crop damage (Lefeuvre et al., 2019). The advances in plant virology are needed mainly for forest tree species that have a current few viral diversity data in their respective forest ecosystems (Rumbou et al., 2021).

In our study, we analyzed the virome of *C. brasiliensis* from Atlantic Forest regions and private properties of southern Bahia using cacao-cabruca AFS through HTS of the RNA samples obtained from vegetative and reproductive organs. Our results showed that the *C. brasiliensis* virome is composed of members spanning almost all members of the Baltimore classification of viruses [+ssRNA, double-stranded (ds)RNA, –ssRNA, reverse-transcribing (RT)-DNA, –ssDNA, dsDNA] that could be classified into at least six distinct families (*Botourmiaviridae*, *Bromoviridae*, *Caulimoviridae*, *Genomoviridae*, *Mitoviridae*, and *Rhabdoviridae*) together with three different unclassified elements. Of note, we successfully reconstituted with high confidence the complete coding sequence of two novel viruses (Carpotroche-associated ilarvirus and Carpotroche-associated genomovirus) and partial sequences from a virus likely representing a novel member of the genus *Gammanucleorhabdovirus*. Proteomics assay from Carpotroche seeds detected peptides from many viral sequences, further confirming the virus presence. Finally, we show the restricted occurrence of viruses from distinct families in exclusive ecosystems and specific organs of *C. brasiliensis*, as well as discrepant abundance among samples. Therefore, our results provide background for a better understanding of the viral diversity in the context of the agroecological interface, such as cacao-cabruca AFS and forest ecosystem.

## Materials and methods

### Sampling design

**FIGURE 1:**
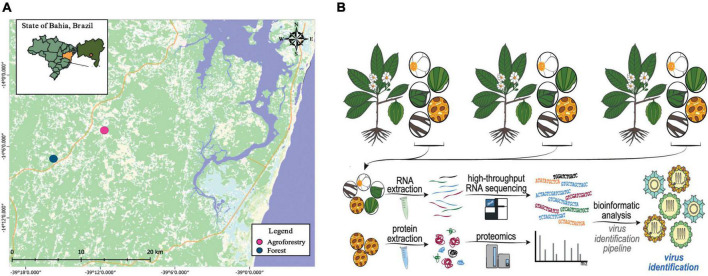
Experimental design of the study. **(A)** Geographic location in the Brazilian Atlantic forest (blue spots) and agroforestry systems (pink spots) where the *Carpotroche brasiliensis* samples were collected. **(B)** Simplified scheme showing the strategy applied in our work, including tissue collection, RNA or protein extraction, RNA deep sequencing, mass spectrometry, and bioinformatics analysis.

Samples from six different organs of *C. brasiliensis* (leaf, flower, flower bud, root, fruit, and seed) were obtained from each individual in June 2014 in Camamu-Maraú Country, State of Bahia, Brazil. All samples were extracted from asymptomatic individuals with no discoloration of any kind, wilting, or necrotic lesions. Sixteen adult trees were randomly selected to compose two sampling groups: eight from agroforestry systems and eight from natural Atlantic Forest ecosystems (Figure 1A; Supplementary Table 1). All samples were immediately frozen in liquid nitrogen and stored at −80°C for RNA extraction at the Center of Biotechnology and Genetics, Laboratory of Molecular Markers, Universidade Estadual de Santa Cruz (UESC), Brazil.

### Plant material, RNA extraction, cDNA library preparation, and high-throughput sequencing

Total RNA was extracted from each sample using RNAqueous^®^ Total RNA Isolation Kit (AM1912, Thermo Fisher Scientific), following the manufacturer’s recommendations. The integrity and quantity of RNA were confirmed using the TapeStation Agilent 2200 instrument (Agilent Technologies Co., Santa Clara, CA, United States), considering an RNA Integrity Number (RIN) value above 5. Only RNA samples of vegetative and reproductive organs with integrity and quantity acceptable were used to generate the 20 cDNA libraries. The cDNA libraries were constructed using 10 ng of total RNA and using the NEBNext Ultra RNA Library Prep Kit for Illumina (E7530S, New England Biolabs, Inc., Ipswich, MA, United States) and the NEBNext Multiplex (E7335, New England Biolabs, Inc., Ipswich, MA, United States) Oligos, following the manufacturer’s protocols (Illumina, San Diego, CA, United States). The libraries were quantified with KAPA Library Quantification Kit for Illumina Platforms (KAPA Biosystems, Wilmington, MA, United States) and Agilent 2100 Bioanalyzer (Agilent Technologies, Waldbronn, DE, United States). A total of 20 cDNA libraries (11 from samples of agroforestry systems and 9 samples of Atlantic Forest ecosystems) were sequenced on Illumina MiSeq 2 × 250 bp pair-end sequencing (Supplementary Table 1) to provide biological replicates. Raw sequencing data were deposited at NCBI Sequence Read Archive (SRA)^1^ under project number PRJNA858666.

### Pre-processing, quality control, and transcriptome *de novo* assembly

Trimmomatic software v.0.32 was used to filter out sequences not fitting in following criteria: minimal average Phred score: 33, Leading: 30, Trailing: 30, Sliding window 4: 30, and Minlen: 50 (Bolger et al., 2014). Pre-processed libraries obtained from different plant tissues were used as input for the transcriptome assembly in the SPAdes tool (Bankevich et al., 2012) in two steps: initially, an assembly of the 20 libraries was made using the *-rna* parameter, with all other parameters kept as default; subsequently, contigs that showed sequence similarity to viral sequences in the previous step were used as an anchor for the new assembly, setting the *–trusted-contigs* parameter. An overview of the methods is shown in Figure 1B. We also performed a metagenomic analysis to determine the presence of possible contaminations using the Kaiju platform (Menzel et al., 2016).

### Virome analysis

Assembled contigs were used as queries in the Diamond tool (Buchfink et al., 2015) against amino acid sequence databases to identify sequences of possible viral origin. In this analysis, we considered only the best hit for each contig and with an e-value < 1e-3. The result was further filtered using regular expression and manually inspected to select only hits of viral origin. The contigs with putative viral origin were submitted to CAP3 (Huang and Madan, 1999) and CD-HIT (Fu et al., 2012) tools to remove redundancy and extend assembled contigs. For CD-HIT clustering, sequences with 90% coverage and 90% identity were merged, and a representative sequence was defined. The putative viral contigs were analyzed by the NCBI BLAST online tool (Johnson et al., 2008) to guarantee the last updated version of nucleic acid databases. The sequences were submitted to BLASTN against the *nt* database and to BLASTX against the *nr* database to obtain the best hit, to confirm the results. The contigs that showed characteristics of viral sequences (contig length and hit with viral sequences) were subsequently analyzed in the ORFfinder tool^2^ to perform its structural annotation. Finally, conserved domains were annotated in each contig using the PHMMER tool (Potter et al., 2018) under the following parameters: sequence e-value 0.01 and hit e-value 0.03. For the quantification of viral sequences, we built the transcriptome index using the putative viral contigs that were compared to each library using Salmon (Patro et al., 2017) with standard parameters. Counts were used to estimate the relative abundance at the transcript level through transcripts per million (TPM) and plotted as heatmap using the ComplexHeatmap package in R (Gu et al., 2016). Reconstituted high-quality viral sequences used in the phylogeny were deposited at the NCBI nucleotide database (GenBank) under accessions OL964097–OL964101. All viral sequences assembled in our work are also available in Supplementary Data Sheet 1.

### Phylogenetic analyses

The assembled contigs were translated into amino acid sequences and aligned with the closest viral sequences available in the NCBI protein database (Geer et al., 2010) using the MAFFT program (Katoh and Standley, 2013). The best-fit model was selected for each alignment using the ProtTest 3.2 program considering the Akaike Information Criterion (AIC) (Akaike, 1974; Abascal et al., 2005). Maximum likelihood (ML) trees were constructed in MEGA X (Kumar et al., 2018), considering 1,000 bootstrap replicates. The trees generated were mid-term rooted and edited in Geneious Prime 2021.^3^ The sequences from the viruses common bean curly stunt virus (unclassified, tentative member of the family *Geminiviridae*), peanut clump virus (*Pecluvirus* genus) (NP_620047), and ampivirus A1 (YP_00913521) were used as outgroup for the phylogenies produced.

### Protein analysis and mass spectrometry

#### Sample preparation

Total proteins were extracted from separate pools of whole seeds derived from staminated individuals using a protocol developed by Pirovani et al. (2008). Briefly, seeds were macerated in the presence of liquid nitrogen and 7% polyvinylpolypyrrolidone. In total, 0.1 g of seeds were used for three replications for stages S1 and S2. The samples were resuspended in 500 μL of 1-butanol: chloroform (1:9), and the mixture was vortexed and centrifuged for 5 min at 13,400 x*g* at 4°C. This procedure was repeated two times. Subsequently, the precipitate generated by centrifugation was washed two times with 500 μL of 100% acetone and centrifuged as in the previous step. Finally, the precipitate was washed with 500 μL of petroleum ether and centrifuged for 5 min at 13,400 x*g* at 4°C. After completion of delipidation, the samples were subjected to precipitation and protein extraction (Pirovani et al., 2008), with the modification being the incubation of the samples overnight at −20°C in the washing step with 10% trichloroacetic acid (TCA) in water. The final precipitate was resuspended in 400 μL of urea at 8 M. At the end, the proteins from the samples were stored in a freezer at −20°C until further use. The protein extracts obtained were quantified with the 2D Quant Kit (GE Healthcare Life Sciences, Chalfont, United Kingdom) following the manufacturer’s recommendations. A standard curve of bovine serum albumin (BSA) was constructed, which served as a basis for the quantification of samples of *C. brasiliensis* seeds.

#### Mass spectrometry

The solution containing the digested peptides was desalted using tips with C18 resin (10 μL; Millipore^®^ Ziptips C18). The peptides were eluted in 50 μL of a solution containing 75% acetonitrile, 25% water, and 0.1% formic acid. The peptides were analyzed in a liquid chromatography system (Agilent 1290 Infinity II HPLC) coupled to a quadrupole/time-of-flight mass spectrometer (Agilent 6545 LC/QTOF) (Agilent Technologies, Santa Clara, CA, United States). Samples were separated using a reversed-phase column (C18; AdvanceBio Peptide Mapping 2.1 × 250 mm; Agilent), maintaining a temperature of 55°C. A 20-min gradient was applied with mobile phases A (water and 0.1% formic acid) and B (acetonitrile and 0.1% formic acid). The percentages of phase B along the grid were 5–35% (1–10 min.), 35–70% (11–14 min.), 70–100% (1618 min.), and 100% (16–20 min.). In addition, a final period of 5 min was programmed for column stabilization. Then, the samples were injected into three technical replicates. The samples were injected into the QTOF through an electrospray source, using the Auto MS/MS acquisition mode, with a maximum selection of 10 precursors per cycle. The parameters for the selection of precursors were as follows: threshold of 1,000, 10,000 counts/spectrum, the stringency of 100% purity, a cut-off of 30% purity, peptide isotopic model, charge preference of 2, 3, >3, and unknown. The instrument parameters were set as follows: gas temperature of 325°C, the gas flow of 13 L/min, a capillary voltage of 4,000 V, and skimmer voltage of 56 V. Nitrogen gas was used for the induced dissociation collision. Instrument control (HPLC and QTOF) and parameter configuration were performed using the Agilent MassHunter Acquisition software.

### Identification of virus-derived peptides

The data integration of transcriptomic and proteomic analyses was implemented through mass spectrometry (MS) analysis to discover proteomics from *C. brasiliensis*. The identification of peptides derived from viral proteins was performed from spectral extraction and merging through analysis with Spectrum Mill Proteomics Workbench (Agilent Technologies, Santa Clara, CA, United States) against proteomic data from *C. brasiliensis* seeds. The static modification to carbamidomethylation was set to default, with a mass (MH +) range of 200–6,000 mass–charge ratio (m/z). Retention time tolerance was ± 60 s, m/z tolerance was ± 1.4 m/z, MS noise threshold was set to 10 counts, and charge general was set to default. The data searched were filtered for validation by score threshold with a false discovery rate (FDR) > 1%. The spectral intensity of identified proteins was searched against the viral ORFs analyzed. The MS/MS spectral comparison included four miss-cleavage sites and fixed modifications: carbamidomethylation on cysteine residues (C), differential modifications for oxidized methionine (M), pyroglutamic acid (N-termQ), deamidated (N), phosphorylated S (S), phosphorylated T (T), and phosphorylated Y (Y). To determine the combined score of minimal intensity, 10% with a mass tolerance of 20 ppm was validated and filtered by false discovery rate (FDR) > 1%. All MS files produced in this study were deposited at MassIVE^4^ with the identifier MSV000089145 and can be accessed at ftp://massive.ucsd.edu/MSV000089145/.

## Results

### Metavirome analysis

We deep sequenced 20 cDNA libraries derived from the tissues of *C. brasiliensis* from forest and agroforestry ecosystems (cacao-cabruca AFS), totalizing 28,177,426 raw reads (Figure 1). After pre-processing steps, including quality and length filters, 24,689,370 (87.62%) reads were kept (Supplementary Table 1). Transcriptome assembly produced 281,643 transcripts with N50 of 481 bp and a total number of bases in transcripts of 160,909,318 (Supplementary Table 2). From the total, 184 sequences showed sequence similarity to viral sequences stored in NCBI public databases, of which 136 (75%) showed hits related to elements with non-retroviral origin. Retroviral sequences were discarded from the analysis since they were of low reliability and often are misidentified with transposable elements. As quality control, we performed metagenomic analyses that did not detect sequences from animals, indicating a low chance of contamination from external sources (Supplementary Figure 1 and Supplementary Table 3). After removal of redundancy and manual curation, we selected 30 sequences larger than 400 nt for further characterization. The length of putative viral contigs ranged from 503 nt to 8,695 nt.

### Diversity and distribution of viral sequences

**FIGURE 2:**
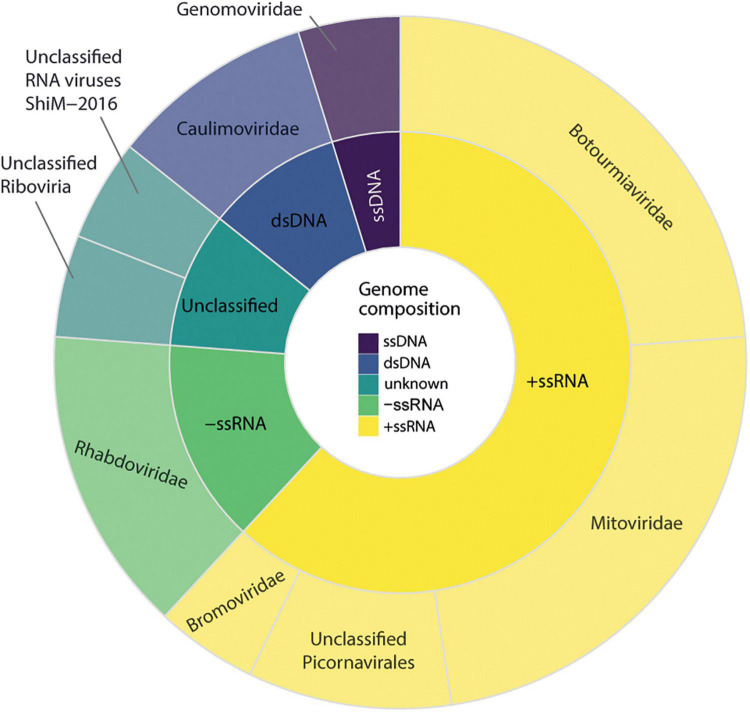
Diversity of viral sequences identified in *Carpotroche brasiliensis* samples. Virus classification was performed based on the information of the closest related sequence present in NCBI databases classified by genome structure and viral family. The diversity analysis only included considered sequences derived from RNA-dependent RNA polymerases, replicase, or polyproteins.

We observed that 30 sequences of possible viral origin are related to viruses from at least six different viral families. Of these, 17 sequences showed similarity to either RNA-dependent RNA polymerases, replicases, or polyproteins, referred hereafter as key sequences, and were considered in the diversity analysis (Figure 2). From the total of non-retroviral viral sequences identified, 16 sequences could be identified by nucleotide similarity, suggesting they are possibly strains of known viruses, and 14 were classified only at the amino acid level (Supplementary Table 4). Overall, sequences showed similarity to closely related viruses from at least six different families or unclassified. Eight transcripts showed similarity to Maize fine streak virus genus *Gammanucleorhabdovirus* (*Rhabdoviridae*, −ssRNA); four transcripts to viruses of the genus *Ourmiavirus*, three to an unclassified virus of the family *Botourmiaviridae* (+ssRNA), five transcripts to viruses from genus *Mitovirus* (*Mitoviridae*, +ssRNA), three transcripts to viruses of the genus *Ilarvirus* (*Bromoviridae*, +ssRNA), and two unclassified sequences showing similarity with picornavirus (+ssRNA); one transcript to viruses of the genus *Caulimovirus*, and one to viruses of the genus *Solendovirus* (*Caulimoviridae*, +dsDNA); one transcript to viruses of the genus *Genomovirus* (*Genomoviridae*, −ssDNA) and other two unclassified transcripts (Supplementary Table 4).

These sequences were detected in the sum of all 20 *C. brasiliensis* libraries analyzed. Co-occurrence analyses indicated that all three segments of a tentative new virus from the genus *Ilarvirus* were detected in 16 samples, while at least two sequences appeared together in 18 different samples. The second most abundant family was *Rhabdoviridae*, genus *Gammanucleorhabdovirus*. In total, the sequences showing similarity to the viruses of the genus *Gammanucleorhabdovirus* were detected in nine libraries of *C. brasiliensis* with a maximum number of seven different contigs in a single library. Then, in decreasing order of abundance sequences presenting similarity with viruses from the families, *Mitoviridae* was detected in 12 libraries, *Botourmiaviridae* was found in 4 libraries, *Caulimoviridae* was found in 3 libraries, and elements of *Picornavirales*, *Genomoviridae*, and unclassified *Riboviria* were detected in two different libraries. Only one contig showing similarity with unclassified RNA viruses was restricted to a single library.

### Characterization of near-complete viral genomes

To increase certainty for high-quality viral sequences, we performed an extra assembly step with Cap3. Four out of the 30 transcripts had their lengths extended, of which three sequences showed similarity to ilaviruses and one was likely derived from an unclassified picornavirus. Through manual curation *via* BLAST (Supplementary Table 4), it was possible to confirm the similarity of these elements to previously identified viruses available in the NCBI sequence databases.

**FIGURE 3:**
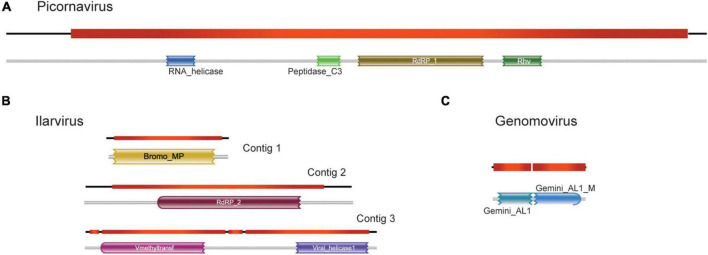
Structural and functional annotation of high-confident viral sequences. For sequences showing similarity to viruses of the families **(A)** *Picornavirus*, **(B)** *Ilarvirus*, and **(C)** *Genomovirus*, the detected open reading frames (ORF) and the presence of conserved domain are shown.

The largest transcript containing 8,636 nt was closely related to Skokie picorna-like virus (SPV), with a size 8,401 bp, 94% nucleotide sequence identity, and >97% of amino acid sequence identity, which is an unclassified picornavirus that infects the mite Dermatophagoides pteronyssinus. Remarkably, we observed very similar patterns of ORF distribution and domain organization when comparing this assembled transcript with SPV (Figure 3A; Supplementary Figure 2), suggesting a close evolutionary relationship. Indeed, they share conserved domains encoded by RNA-dependent RNA polymerase (PFAM PF00680) and capsid protein (PFAM PF00073) of viruses from the order Picornavirales (Figure 3A). For sequences presenting similarity with the elements of the family Bromoviridae, the Contig1 (2,442 nt) was closely related to the phytopathogenic virus Lilac ring mottle virus (LRMV), with 2,287 nt and 73% identity, which belongs to the genus Ilarvirus. Both sequences also share the same pattern of ORFs, although the main ORF in Contig1 is 11 amino acids longer than the main ORF of LMRV (Figure 3B; Supplementary Figure 3). Domain analysis indicates that this major ORF encodes for a Bromovirus movement protein (PFAM PF01573), increasingly the possibility of functionality (Figure 3B; Supplementary Figure 3). Contig2 (2,954 nt), on the other hand, was most similar to another phytopathogenic ilarvirus Citrus leaf rugose virus, with 2,990 nt and 72% identity. Besides having little difference in length, they also had similar ORF patterns (Figure 3B; Supplementary Figure 3). The major ORF differs from one other by only 36 bp, and both code for the RNA-dependent RNA polymerase enzyme (PFAM PF00978) (Figure 3B; Supplementary Figure 3). Contig3 (3,467 nt) was closely related to a phytopathogenic ilarvirus, Tomato necrotic streak virus (with 3,378 nt and 68% identity with segment RNA 1). Structural annotation shows that the longest ORF in both sequences differs only by 4 nt, which was only identified at the amino acid level as the replicase protein. Domain analysis also shows that both longest ORFs share methyltransferase (PFAM PF01660) and helicase (PFAM PF01443) conserved domains (Figure 3B; Supplementary Figure 3).

We also selected one transcript showing similarity to that of the family *Genomoviridae* to undergo further structural and functional annotations. This transcript was chosen because the sequence recovered corresponds to an intact replicase with a length consistent with genomoviruses. This transcript (943 nt) showed the highest identity to chicken genomovirus mg4_1247 (complete genome: 2,142 nt; replicase: 1,008 nt, 62% identity) (Figure 3C; Supplementary Figure 4). Domain analysis revealed that both sequences present a conserved Gemini_AL1 domain (PFAM00799) (Figure 3C; Supplementary Figure 4).

### Phylogenetic analysis of Carpotroche-associated viruses

**FIGURE 4:**
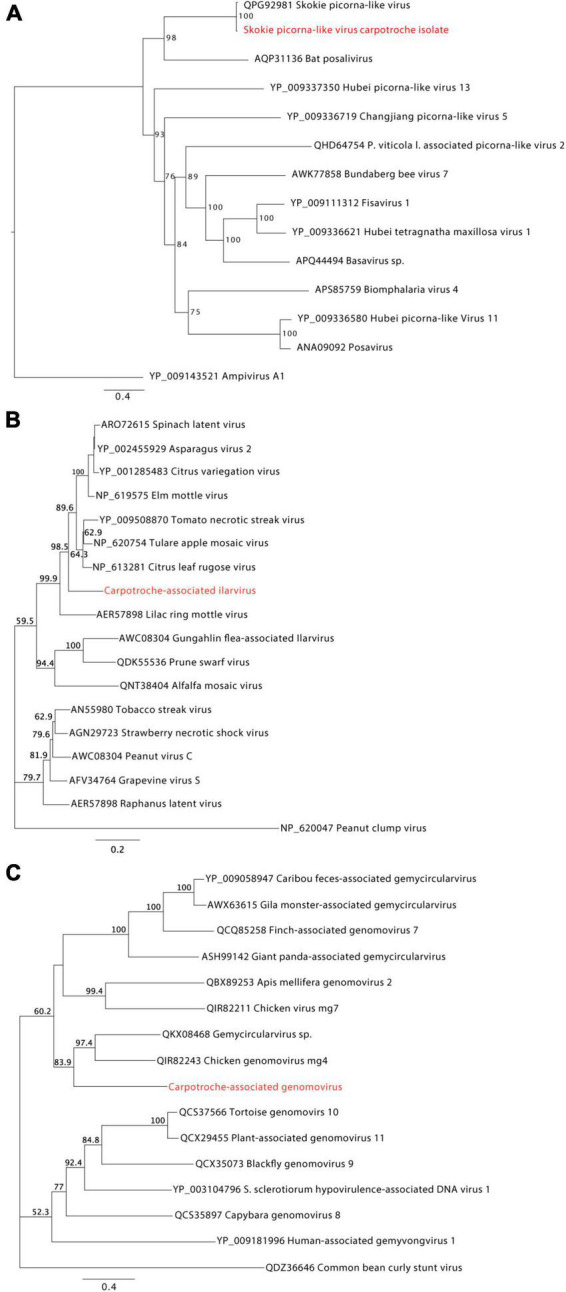
Phylogenetic analyses of high-confident viral sequences identified in *Carpotroche brasiliensis*. Phylogenetic trees inferred by maximum likelihood for transcripts associated with *C. brasiliensis* and showing similarity to **(A)** picornaviruses, **(B)** ilarviruses, and **(C)** genomoviruses species. RNA-dependent RNA polymerase or polyprotein aminoacid sequences were used in the analysis. Branch support was calculated using the bootstrap method, requiring 1,000 pseudoreplicates. Highlighted regions in red correspond to the assembled sequences obtained in our work.

We selected putative viral sequences with near-complete or complete coding sequences according to the closest relative virus in public databases to perform further phylogenetic characterization. Therefore, here we assessed the phylogenetic relationship of the three viral transcripts with their closely related viruses from the genera *Picornavirus*, *Ilarvirus*, and *Genomovirus*, respectively (Figure 4). The phylogeny of the sequence corresponding to a virus of the family *Picornaviridae* confirmed the similarity observed by local alignment with the Skokie picorna-like virus with 100% of bootstrap. This sequence was also related to the Bat posalivirus, suggesting that the assembled transcript belongs to the genus *Picornavirus* (family *Picornaviridae*) (Figure 4A). This virus was named Skokie picorna-like virus Carpotroche isolate. The transcript showing similarity to viruses of the genus *Ilarvirus* was close to the clade of plant ilarviruses containing viruses of the species *Citrus leaf rugose virus*, *Tulare apple mosaic virus*, *Tomato necrotic streak virus*, *Spinach latent virus*, *Asparagus virus 2*, *Citrus variegation virus*, and *Elm mottle virus* (Figure 4B), thus we can infer that this sequence is from the genus *Ilarvirus* (*Bromoviridae*) and the putative virus was named Carpotroche-associated ilarvirus. In the phylogeny of the element previously associated to *Genomovirus*, we observed that the sequence grouped with the virus chicken genomovirus mg4, and Gemycircularvirus sp. with a bootstrap value of 97.4%, indicating that this species belongs to the *Genomoviridae* (Figure 4C). This putative new virus was named Carpotroche-associated genomovirus.

### Characterization of a tentative new virus from the genus *Gammanucleorhabdovirus*

**FIGURE 5:**
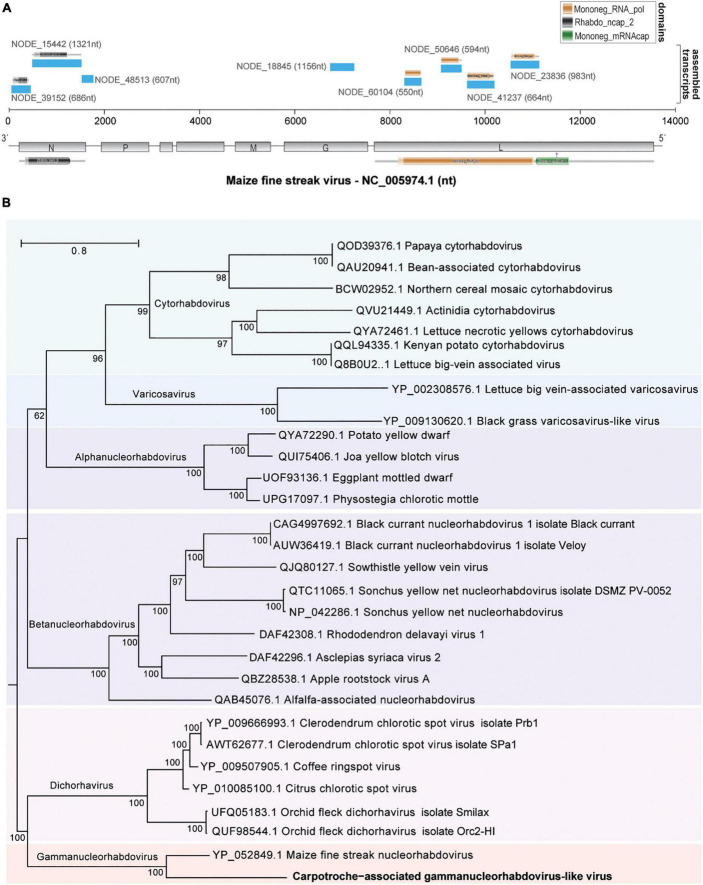
Characterization of a putative gammanucleorhabdovirus. **(A)** Distribution of assembled transcripts along the genome of the closest relative virus, Maize fine streak virus (MFSV). Conserved domains are indicated above each assembled transcript or below the MFSV genome. **(B)** Maximum likelihood tree containing sequences from viruses of the subfamily *Betarhabdovirinae* (*Rhabdoviridae*) based on the largest fragment, 983 nt, derived from L (polymerase) gene.

Metavirome analysis revealed a considerable number of viral transcripts likely derived from a plant-infecting rhabdovirus related to the viruses of the *Gammanucleorhabidovirus* genus. Indeed, 8 out of 30 viral transcripts presented similarity identity values at the protein level, ranging from 32.54 to 71.88%, with Maize fine streak virus (MFSV), the unique known member of the genus *Gammanucleorhabdovirus* genus accepted by ICTV (Figure 5A). These transcripts ranged from 550 to 1,321 nt totalizing 6,561 nt with an average size of ∼820 nt and were distributed along the MFSV genome. Six out of the eight contigs presented conserved domains commonly identified in rhabdoviruses, including MFSV (Figure 5A). Of note, we identified one transcript of 983 nt showing similarity to the RdRp gene, containing a Mononeg_RNA_pol (PF00946) domain. We took advantage of this transcript likely derived from viral polymerase to assess the phylogenetic relationship of the tentative virus with MFSV and other rhabdoviruses. According to our maximum likelihood tree, we observed the presence of six main clades, which refer to different genera of the subfamily *Betarhabdovirinae*—*Rhabdoviridae* (*Cytorhabdovirus*, *Varicosavirus*, *Alphanucleorhabdovirus*, *Betanucleorhabdovirus*, *Dichorhabdovirus*, and *Gammanucleorhabdovirus*) (Figure 5B). The transcript assembled clustered with Maize fine streak virus in a clade-specific manner with a bootstrap of 100, suggesting that the sequence probably belongs to a species from this genus (Figure 5B). However, since we were not able to reconstitute the complete genome of the virus, we named the viral species as a Carpotroche-associated gammanucleorhabdovirus-like virus.

### Ecosystem distribution and organ tropism

**FIGURE 6:**
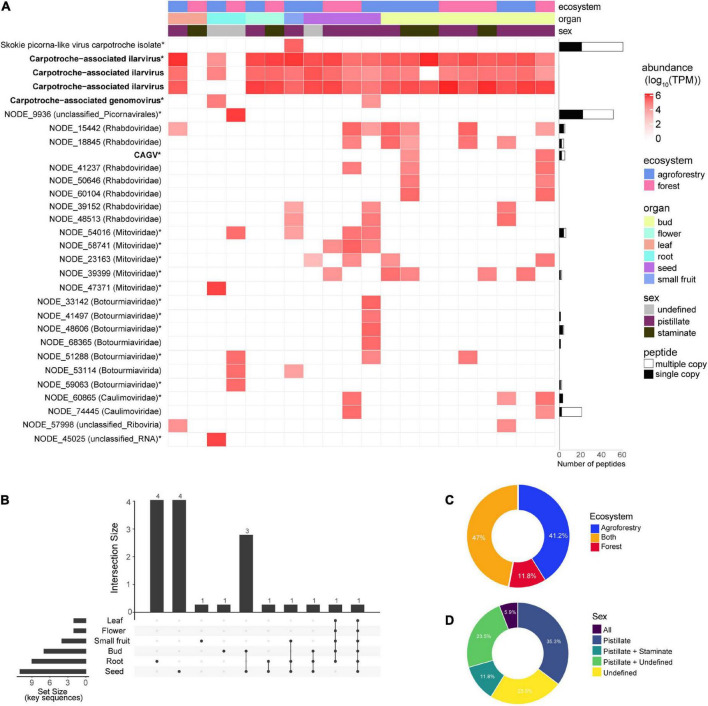
Abundance and distribution of Carpotroche-associated viruses. **(A)** Heatmap was constructed based on the relative abundance of each viral transcript identified classified by ecosystem, tissue, and sex. Quantification was computed using transcripts per million (TPM). Horizontal bars indicate the abundance and uniqueness of peptides derived for each of the assembled transcript key sequences and are indicated with *. CAGV, Carpotroche-associated gammanucleorhabdovirus-like virus. The presence of key viral sequences by plant tissue **(B)**, ecosystem **(C)**, or sex **(D)**.

Since we deep sequenced the RNA of several tissues from vegetative and reproductive organs of *C. brasiliensis* originated from forestal ecosystems and agroforestry, we decided to explore the tropism of the identified viral sequences among the individuals assessed in this study. We also analyzed the relative abundance of viral elements detected in organ samples for each ecosystem. We noticed that the seed was the tissue with the greatest diversity of viral sequences, with the presence of 20 (11 key sequences) out of the 30 viral sequences identified in our study (Figures 6A,B). The libraries of floral bud, root, and small fruit also showed high percentages of viral diversity, with 18 (eight key sequences), 10 (eight key sequences), and 8 (three key sequences) viral sequences, respectively (Figures 6A,B). However, we acknowledge that these data have to be interpreted carefully, since we analyzed only one library of small fruit while we assessed nine libraries for floral buds. Besides, in one of the libraries from *C. brasiliensis* leaves, we were not able to detect any sequence of viral origin (Figure 6A). Of note, over half of the key sequences (10) were organ-specific, while approximately 22% were found in at least three different organs (Figure 6B). Surprisingly, only one viral sequence of Carpotroche-associated ilarvirus was identified in all tissues assessed (Figure 6B).

Since tissue tropism analysis indicated seeds as containing the major number of viral sequences, we decided to perform a proteomics assay for the sample derived from whole seeds collected from staminated individuals to provide an additional level of confidence. From the 17 putative viral family elements identified in seeds, we were able to detect peptides for nine of them. We also detected peptides from NODE_15442 (*Rhabdoviridae*), NODE_18845 (*Rhabdoviridae*), NODE_68365 (*Botourmiaviridae*), and NODE_74445 (*Caulimoviridae*) sequences previously identified in other organs (Figure 6A—horizontal bars; Supplementary Table 5).

Relative to ecosystems, the abundance of viral elements in equivalent tissue samples is slightly higher in specimens originating from the cacao-cabruca AFS (41.2% of total sequences) when compared to the forest ecosystem. Viral sequences detected exclusively in the cacao-cabruca AFS were Skokie picorna-like virus isolate Carpotroche, Carpotroche-associated genomovirus, botourmiavirus (3), non-classified sequence (1), and mitovirus (1). In contrast, one sequence related to element from botourmiavirus and one related to unclassified picornavirus were identified exclusively in samples obtained from the forestal ecosystem (Figures 6A–C).

Regarding the reproductive organs, in both ecosystems, samples obtained from stamen tissues show a lower abundance and diversity in viral sequences compared to those obtained from pistil tissues (Figure 6D). While we noticed species that were organ-specific, none of the viral sequences were exclusively found in samples derived from staminate plants (Figure 6D).

## Discussion

In this study, we identified viral sequences representing the *C. brasiliensis*-associated virome in native forest and cacao-cabruca AFS. Through HTS technologies associated with proteomics assay, it was possible to perform an in-depth analysis of viruses putatively associated with this plant, expanding our knowledge about environmental virology and describing the profile of viral infection among samples and ecosystems. Also, we enlightened the abundance and distribution of virus infection per vegetative and reproductive organs of *C. brasiliensis* and the composition of viral communities per sampled landscape.

The patterns of diversity, abundance, and virus tropism between vegetative and reproductive organs observed in this study can be related to the host preference of a virus, as well as to the immunological capacity in different cell types and tissues of the host (Navarro et al., 2019; Keesing and Ostfeld, 2021). Therefore, this can impact the amplification effects arising from increased transmissibility among hosts or dilution effects of infections in agricultural environments or in natural ecosystems (Keesing et al., 2006; Keesing and Ostfeld, 2021; Susi and Laine, 2021). Interestingly, we identified *C. brasiliensis* putative viruses related to two different families that have species described to infect different plant hosts of forest trees or crops, i.e., *Ilarvirus* and *Mitovirus* (Pallas et al., 2013; Rumbou et al., 2021). Of note, the highest diversity of these virus sequences was observed in reproductive organs. It is likely that viral exchange exists at the agroecological interface through the transmissibility of viruses detected in the reproductive structures of *C. brasiliensis* during pollination processes and dispersal over longer distances due to animal intermediation (Marangon et al., 2010; Zucaratto et al., 2010). Viruses with these characteristics can also mutate acquiring to the possibility to infect unrelated alternative hosts in the same ecological community, such as detected in cacao-cabruca AFS or forest ecosystem (Elena et al., 2009).

We observed widespread infection in *C. brasiliensis* by a tentative new virus of the genus *Ilarvirus*. Viruses of this genus can infect woody plants, such as *C. brasiliensis*, as well as fruits, vegetables, and forages of economic interest, such as peach, apricot, tomato, apple, vine, cucurbitaceae, banana, and alfalfa (Pallas et al., 2013). Ilarvirus infections range from no observable symptoms to the occurrence of leaf mosaics, structural malformations, abortive flowers, and atrophy in fruits and seeds, and in some cases, may lead to host death (Girgis et al., 2009). Contigs belonging to detected ilavirus (Carpotroche-associated ilarvirus 1, 2, 3) presented homology with three different viruses: citrus leaf rugose virus (CLRV), lilac ring mottle virus (LiRMoV), and tomato necrotic streak virus (TomNSV). CLRV can infect a wide range of citrus hosts with induction of milder symptoms, while LiRMoV can induce leaf deformation and reduce leaf size, ring spots, and line patterns (Sharma-Poudyal et al., 2016; Zhou et al., 2020). The infection with LiRMoV was observed in herbaceous plants in the experimental conditions, although it is possible that cryptic infection may exist in crop plants for many years (Sharma-Poudyal et al., 2016). On the other hand, TomNSV infects the *Solanaceae* and *Chenopodiaceae* families, producing serious damage to the leaves (Badillo-Vargas et al., 2016). However, we did not find any symptoms in *C. brasiliensis* individuals sampled.

Virus diversity observed in organs suggests an established viral community in *C. brasiliensis in situ*, with possible transmission between ecosystems *via* arthropod vectors, such as for the virus alfalfa mosaic virus, where thrips and aphid contamination is mediated by feeding on pollen grains or *via* direct contact with infected pollen, seeds, or fruits (Pallas et al., 2013; Silvestre et al., 2020). The arthropod vector-mediated contamination may also be related to the transmission of virus of the genus *Gammanucleorhabdovirus* (*Rhabdoviridae*), with different sequences detected in the samples of leaves, fruits, seed, and flower buds obtained from *C. brasiliensis*, showing similarity to the segments of MFSV, such as nucleocapsid protein (N), glycoprotein (G), and polymerase (L) (Dietzgen et al., 2020). Plant viruses of the family *Rhabdoviridae* have global circulation with damage to diverse commodities, including maize and wheat crops in South American countries, such as Argentina and Peru (Willie and Stewart, 2017; Maurino et al., 2018; Dietzgen et al., 2020). Infection symptoms by these viruses include yellowing, chlorosis, and streak formation on leaves, as well as dwarfism and leaf deformation. Although the specific vector of MFSV, the leafhopper *Graminella nigrifrons* (*Cicadellidae*) has been reported only in the United States and the Caribbean. These insects belong to the families *Cicadellidae* and *Delphacidae*, which have the potential to vector rhabdoviruses that are commonly found infecting grasses in Brazil. Although only partial sequence assembled, after further validation, this find could represent the first report of a virus from the genus *Gammanucleorhabdovirus* in South America (Todd et al., 2010; Oliveira et al., 2013; Dietzgen et al., 2020).

Viral sequences found in the root of *C. brasiliensis* may be associated with variation in soil biodiversity between forest and agricultural ecosystems (Pacchioni et al., 2014). Soil particularities between ecosystems (chemical composition, temperature, oxygenation, and organic matter) may influence the diversity and abundance of the microbiome, with a potential to be vectors or virus hosts in the soil, making the root susceptible to infections from different sources (Rodelo-Urrego et al., 2013; Souza et al., 2016; Tripathi et al., 2016). We identified sequences from viruses of the families *Mitoviridae* and *Botourmiaviridae* in seeds, floral buds, and roots. Mitoviruses have genomes containing a single ORF that encodes to an RdRp that presents genetic code specific for mitochondrial genomes, and possibiy the virus uses exclusively the mitochondrial machinery of the host in their replication cycle (Hillman and Cai, 2013; Nibert et al., 2018). Mitoviruses can cause latent infection in hosts and are thought to be transmitted by cell division or through the dispersal of spores (Nibert et al., 2018). In phytopathogenic fungi, the infection can result in fungal hypovirulence, revealing the potential of mitoviruses for use as biocontrol agents (Wu et al., 2010; Xu et al., 2015). Evidence shows that mitoviruses possibly adapted their genome for a cross-kingdom transmission due to the co-evolutive relationship between fungi and other organisms, such as flowering plants (Nibert et al., 2018; Fonseca et al., 2021; Wang et al., 2022). Viruses of the family *Botourmiaviridae* can be grouped into six genera. The first one presents three genomic segments and infects the cytoplasm of plants, whereas some other members infect filamentous fungi (*Ourmiavirus*). The second genus comprises viruses with a single segment and able to infect fungi and plants (*Scleroulivirus*), and the viruses of the four other genera (*Botoulivirus*, *Magoulivirus*, *Penoulivirus*, and *Rhizoulivirus*) infect mainly fungal hosts (Ayllón et al., 2020). Recently, representatives of the families *Mitoviridae* and *Botourmiaviridae* have also been detected in mycorrhizae, which are responsible for forming inter- or intracellular structures in roots of more than 90% of plant species, which assist in nutrient cycling, water uptake, and disease resistance (Bonfante and Genre, 2010; Sutela et al., 2020). The results found in our study indicate that the presence of viral genomes from this family can be ubiquitous in plant samples and may indicate a possible interaction among virus, plant, and fungal cells.

We also observed the presence of picornaviruses in the vegetative organs of *C. brasiliensis*, which had the closest hit with a polyprotein of Biomphalaria virus 2 (coverage of 95% and identity of 54.3%). In the phylogenetic analysis, the studied virus was grouped with Pittsburgh sewage-associated virus 1, a virus that was isolated from an urban sewage sample with an undetermined host (Cantalupo and Pipas, 2018). Biomphalaria virus 2 was originally identified in the microbiota of health snails *Biomphalaria glabrata* and *B. pfeifferi*, vectors of protozoa from the genus *Schistosoma* (Palasio et al., 2021). Previous studies using RdRp indicated the phylogenetic relationship of Biomphalaria virus 2 with viruses of the family *Secoviridae*, the only one from the order *Picornavirales* that has been identified to be infecting plants to date. Therefore, the presence of a picorna-like sequence only in the *C. brasiliensis* root sample obtained in the forest ecosystem could be driven by the presence of snails in this region. A second virus showing similarity to picornavirus (Skokie picorna-like virus carpotroche isolate), specifically to Skokie picorna-like virus, was found in our analysis. Another virus close to the studied picornavirus in the phylogenetic tree is Bat posalivirus, originally isolated from bat feces in Cameroon (Yinda et al., 2017). Coupled with the fact that the isolate of Skokie picorna-like virus is present only in fruit samples, this information indicates the possibility of this virus infecting *C. brasiliensis* to be transmitted by a vector. Considering the pathogenicity of picornaviruses in several species and the economic impact of severe vector-mediated plant diseases, the identification of this viral sequence is of central importance in the investigation of the *C. brasiliensis* virome (Jia et al., 2018).

Among the DNA viruses detected, a putative caulimovirus was detected in seed and floral buds. Viruses of the family *Caulimoviridae* have a circular dsDNA genome, with no envelope and a reverse transcription (RT) step in their life cycle, and are transmitted *via* arthropod vectors with several representatives considered important pathogens for a wide diversity of monocot and dicot plants, including apple, citrus, banana, cocoa, grape, cassava, rice, potato, corn, papaya, soybean, tomato, and others (Bhat et al., 2016; Teycheney et al., 2020). Some family members also possess the ability to integrate part of their genomes in the form of minichromosomes into the host genome during their replication cycle (Diop et al., 2018). Particularly for *T. cacao*, the studies mostly focused on symptomatic infections that affect the economic potential of the production with the genus *Badnavirus*, and correlate to the outbreak of Cacao Swollen Shoot Virus Disease (CSSVD) that started in 1922 in West Africa and is still devastating cacao production in Eastern African regions (Mondego et al., 2016). Thus, 10 species of viruses were described in West Africa or in some Caribbean islands, and most recently, one isolate of CaMMV-BR-like virus was identified in the Bahia state, Brazil, and also one species (*Cacao bacilliform Sri Lanka virus*) from Sri Lanka (Muller et al., 2021; Ramos-Sobrinho et al., 2021). It is likely that the caulimovirus found in *C. brasiliensis* is an EVE, given the sequence detected has a more likely link to the Aristotelia chilensis virus 1, an endogenous virus corresponding to the genus *Petuvirus* (Villacreses et al., 2015). DNA viruses of the family *Genomoviridae* were also detected in *C. brasiliensis*. This group of ssDNA viruses is considered a sister group of the family *Geminiviridae* and has the potential to cause fungal hypovirulence, identified in different environmental samples associated with plants and animals, including in Brazil infecting common beans and citrus (Lamas et al., 2016; Chabi-Jesus et al., 2020; Fontenele et al., 2020; Varsani and Krupovic, 2021). Phylogenetic analysis of the putative viral sequence suggests its association with viruses from the family *Genomoviridae*, indicating a close relationship to chicken genomovirus mg4 and other viruses of the genus *Gemycircularvirus*. While the first is pathogenic to birds, the latter can infect the phytopathogenic fungus *Sclerotinia sclerotiorum* and decrease its virulence (Varsani and Krupovic, 2017).

To corroborate the viral sequences present in our samples, we performed a proteomics assay to explore the presence of viral proteins. Mass spectrometry-based proteomics for viral infection analyses employed in this study is recognized for both structural virology and virus–host interaction studies (Dülfer et al., 2019). The detection of the charge state spectrum at high resolution allows the investigation of size, mass, stability, and shape of viral protein complexes (Uetrecht and Heck, 2011; Dülfer et al., 2019). Thus, the strategy to compare peptide sequences in different MS spectra readouts is ideal to detect the direct presence of viral proteins. In our analysis, we detected peptides derived from 13 viral sequences, eight of them representing key sequences from the variants of known viruses or viruses likely belonging to novel species. In seeds, the same organ for which we performed proteomics assay, we were able to detect viral peptides for 9 out of 17 sequences identified in RNA sequencing analysis.

## Data availability statement

The datasets presented in this study can be found in online repositories. Specific repositories and accession codes for each dataset are specified at “Materials and methods” section.

## Author contributions

EA and FG: conceptualization, methodology, and supervision. ÍL, PF, RO, LV, and FB: formal analysis. EA, FG, and CP: resources. AV, ÍL, PF, FG, and EA: writing of the original draft preparation. AV, ÍL, FG, and EA: reviewing and editing. FG: funding acquisition. All authors have read and agreed to the published version of the manuscript.

## Footnotes

1
https://www.ncbi.nlm.nih.gov/sra

2
https://www.ncbi.nlm.nih.gov/orffinder/

3
https://www.geneious.com

4
http://massive.ucsd.edu
