# Supplementary material for: Expanding the environmental virome: Infection profile in a native rainforest tree species
Source: Front Microbiol. 2022 Aug 4;13:874319. doi: 10.3389/fmicb.2022.874319 (PMC9387356; doi:10.3389/fmicb.2022.874319)
Supplement: Supplementary file 1 [file Data_Sheet_1.DOCX]

>Carpotroche-associated picornavirus, polyprotein

TGTTTTTTTAATGATACGGCGATCACCGAGCACTACTCTCTTTCTCTACTTTTTTTTTTTAATGATACGGCGACCACCGAGATCTACACTCTTTCCCTACACGACGCTCTTCCGATCTCTGTATTTGAAACGATAAGTCTTTATGTGCATTCATATTCTATAACTAATTAAATAAATCTGTCTGGAATAAACATTCAGAGGTTTGAGCAAGTCTCTTCACATGAATGAATTCGCAATAATTTTGAAGTAACCATTTTACAACGGAATGAAATTGAAAGAATTGTTTTTCAACAATTAAAATTAAAATTATTTTCTTGAATATTTGATTTAATAAAATTTTAAAAAGATTCTGGATTAATGAGTTTCTTAAGTAGAAACATAACTAACGGTTAAATTGAAAACCACCACGAGGTGTGGTTCCAGTAATATCCATTTTATCATCAAATGGTTTATATGTCACTACCGGTCGTGGTACAAATAATCGACAATCATCCCCCATTTTAAGATAAACTTCTAAATATTTGCCAAGTGGAGCCTCACATCTCAATGTAACTGAAAATGGTAGATTTTGCAACCCATCAAATAAACTATTGGACACTGGCAAGAAATTCAAAATTGTATTAAATGGTATGTTAACATCAATATACTGATAAGCATTTGCAATCGGATACAAGTTTTCACAAGGATTATACCGATTAGCATCAACCAAATAAGTTTTATATGAAACCTTATCATTCTTTGTTTCAATAATTGTGGCATTAGTTGTTTCCAAAATAACTGGTCCCACAGAAGGAGAAAAATTAAGAGACATTTCAACCGGAGTTGTATGTTTAACAAAAATTCTCATTTGCAAGTGTCCTGCCCAATATTGGAAAGCATCACGCCACAACCCAGGCAATGTTGGAATGAACACAGTTTCATTAGTATCATGCCGAAAAGTTACATCAACACGTGTGTATCTGCGGAAAACTTCACCAAAATTATTCCATGTGTATTCAAATTTTTCACCCAACTTAAGAATACATGGCTTAGGAACCACAATTGGTTGATAATCATCAACAGGAACAGCTGTTGTTGTTGTAAAATCATCAACTCGTTCTTCTTGTTTTTGTACTTGTGATGTTGTGTTTGATTGTGCTTCATATTCATCTTTATCTGGATCATCATTTTTCTTCAAAGCTGTTGCTTTAATATTAATTCGGTGTGGCACATAAGGTCGAAATTCACGCAGTTTAACGTTTTCACAGCTGGCAAAAACAATAACCTGAACACTTTGTGCTACTGATTGTGGACATTTAAGTTGATTAGCAACCGTTATTTGAATGGATCCCATTTCTTGATTGTATAGATCATAAGAATCTAATCCTTCTTTAACTTTTGACATGTTGAAAGCCGAAATAAGATATTCTGTGGGAGCATTGAATGGCACAACTATTTGTACTATTTCTGATCCTGATGTAAAATCCATAATTTGATTCCGATAAATCGTATCTTGTAATTTTGTAAATGGTTCCGCCCCATATGCTACTGATGCACGCAATCGACCTGAATGGAAATCACTCATAACACACAAAAATGTGAATTTAAAATCTGCTCTCCAAAACATGGCACAATTAAGAGGAAAACAACATGATGGTGTCCATATTCCTTTAACAGCTTCTGGATTTGACCAAGACAAAATAGAATTCAGTGGAATAGACATTTTCACATCACCCACATTATCCTGAGGTGTCCAAGTAAAAGATCCAAGAACAGTCGGTCTGTTACAATAAGCTTCAACCGACATATCGGCATTCATTCCTTCAATCTTGTGTGCTTGACGATGCATTTCTTCTGGATGGTGAGCTAAGGCAACTGATGTTGAAATGCCTACTGTTTTTGATCTTGATGGAAAAACGTCATAAACTGGTAAACTACCAGAACATACTGGGGGGTTGTCCAAAGGCAATGCAGAAACATTTGGATTAGCTTCTACACTAGCAGTTGCTGATTGTTTATTTGAAGTATTCAAAGGAACATCACCAACAATGTTGGACAACTGATAAGTATTGTTGGTAGATATTCTATTACCTTGAGCTTCAAATTCATCAGGTTGTGGTGATGGTTCCATGTCAGGAACTGGACGTGTTAGAAGATCACCTGCCAATCTTGTTCCACTTGATGCATATGGTCGTGGAACTCTACATTTAAGATTTGGAAATTTAGTAAAAACAGAAACAGTCACTTCACTTGCTCCGTCCTTACCTGTGTTGAGAGGACTCAAAACACAAATATCCAATGTTGCAAAATTTTCTTTCTTTGTGCTGATTATCCAATCTCTGAACCAAACAAAAGGCAATCTCAACGTTGCTGTTGTTGATGTGTTTGGCGACATTAAAACATGATTGTAAAATGTATATGCAAATGCATTAGTACTATAACGATAACCACCGATATTTGACTCTGCTGCATCCATTAACTTAGCCGGAACCAATACGGCAACAATAGTACCCTGATGAAATGGGGTTCCTGCTATTTGTATTGTAACTTCAACATCATGTCGTAAATACAAATATCTTTCAAAAGGCATGTTTTGTAAATTTTGTTGTTGACCTTTTTCCAAAGCACCAAATGGCAATTCCATACGCAAGAGCGATTTCTTGGCTTCATCATTTAATGTCCATTGAACATCAGCTCGGTGAATTAATGAATCAGTCCCTAGATCCAAAGTTCCCGGAGCTTCAGTTAATCCAGAATTTCCAAATACCATTGAATTAGTAGTTGTTCCATCTTGTTCTTCAATCTTCGAATCAATAACTTGCGTCAACCCCACACGTGATTGAGCACGAAACCAATCTTCTGTGCTGTTTGCATTCCGATGTGCAGCAATACTTTGCATTTCCAAATAACTAGGATATTCAATAGGATGAACAACATTAATTGCATTAAAAGCCAACAAAACTGAGTTTTTATAAGCAATATGAAAATTTTCATCCCATAATGCCGACAAATAAATACATGCTTCCACAACAGTATTCAAATCAACATTGTTAGTTTTGGTCCATTGTACAGTTTGCCACAATTGTTGTTGTTTAGGAGCGCCAACATAGGTCATCATACCGTCGACTTGAATAAGACGAGGGTGAGCTCCAAGAAATGTAATATCTTCAAAGGTTCGAAAATCTGATGTTAATTCTTTGTCTTTGATATCACTGGTGTATTCTTGTCCAATATTTCGCATTTGTGCTTTGATAACAAGAGGATTAAAATCTACTCTATCACTGACAGAGAGAATATGATCATCCCCTAAAACTTTCAAACGACATTCTTTATCGAATGATTTTGTTGAAAAAGCACTTGCAAATGCGTATCTAAAATATAATTCATTCACAAAGCAGTTAATAACCGATGTTAAGAAGCATCCAGATGTATGCATGCAGTATGTTTCAATTAACTCCGATTTAAATTGTAATTTAGGATTAACAGCTTCATGTTTCCATAAATATTCACAATTGTTTTCATCTTGTTCCGATAATTCAAAGAAAATTTCATAAGCAGCTCGTTGGAATTGAACATGATAACAAAAATCATAATTCTTATAGTCACCAGCAACAAAACGTTTATTACCAGTTGATGTTAAATAAGAATAAATGTTATCCATGTCTGTCGAATATTGATTCATTCCAATTGCTGATGGTGTTGCATTTGATGAATTATTAATACGTGCCAATAAAATTCCGAATTTAATTCTAAAAGCCACCAATGATATAACATCATTACAATAAATGGCTCTCGTCCTTGCTTCTTCAATTTTACTGGGTGATACAAGTTCATCTTTCCAATAAACGACAAATCTGTTGTTAGGTGTTACACCGCGTTTCATTTCATCCAACCGCTGTTGTACTGCTGTTCTAAACCATGGTTCAATTTCAACTTCACCTCCTGGTCTGATCTTAAAACAACCAGTTTTCCCCATGGAATTAATCATTTTACTAACAGGATATCCTGGTGATGTTTTCAAATTCAATGATGACAAATATCCTGGCAAACCTAATATTGCTTCTTCTATTGTTAGATCGCGCAAAGCGTATGTTTTATCAACATTACGTCGCCAGTTTTTCAACATGGTTCTTTTGCAACTCTCCAAACGTGAAGAATCAATAACACTATCATTTACTTTGGAAATGCGTTCAATGAAACTTTGTGAAGGACTATTACGTGTTCTACTATCACCTTCTGCCATTATTGATGGTTGTTTCATACTTTGTACTGGTAAATTCCCATTTAATAATGATGGTTGTAATTTTGTTCTTGTTGGAAAGAACATAATTTCAGATGCTGGCAATGATGTTGTTTTAAGGATGTTTGAAACTGGGAATTCAATTGGTTTTTCTTTTGGTGAAAATGGACACCACGATTGTGCTTCCATTTTAACATCTTCATCAGTTGGAACAATACAAAGAGCATCTTTGAGGGCTTTAATATCCTCACAAGTCACTGCTACTGCACCGCCCATAGGTTTGGATTTGTTTTTGTTTCCACATACATGAATTCCAATTATTTTTGCTGGAGCAATTTTATCTGCACTACGTACAATTGCACCACAATCTCCTTTAACTGTTTCAGCATGATAACAGAACATTTTGCTAGGTTGATGTTTAGCAAAAGCTGAACTGTATGACCAACCATCAGTTAATCTTACCGTCAAATAGATCCGTTCATTAGGTTTTTCAAATGTTGTTTGCATAGTATCAATATTTCCAAGTTCAGAATTACTGATAAACTTTGATGAACGATCTTTTGTGTTTGGAAAAGAAGGACAACTCACTCGAAACAATGCCAAGTCATTTTCAGGATCAATAACGACATCTTCAGGACTAACCTTTGTTTCATAAGTGTTACCAGAGAAACATAGGGTCACATCAGATCTCTTCTTAATACCTTCCCATAAATGGCAAGTTGTCAAAACCAAACGTTCACCAATATTAACACATAATTGTGTCCATATGTCTTCTTCTTGTACCATTATTGTTCCAACAATTTCAGTTGATGAATGTGCTTCGAATTTATTAAGACGTGTGATTTTCCGTTGTCTTTGTTTAACATCCCTTTGTTTGCGTCCTGAATCTGTTTCGCCTTCAAATGTGTTGTAATATGACAAATGTTTTTCATAAGCCGCTTCTTCGCAGTACATAAGGTAACCGCAAAAGATTAACCAAAGGATTATCCACCAATTGAAATTGGAAGGATCACCAATTGTTTTAACAGCCTTTATTCCAAAGTTCTTTACATTCTTCAAAATTTTGATGGTATAACCACTAATCGTTGAATAAGTCCATTGATAATGATTATTAAATTTTGAGATTGCATATTGAATGCTTAATGGAAGTCGATCTTCCCATGTTGCCAACATTGCCAAACGACGTTGGATTTCCTCATCTGTTATGTTTTCAGGCACAACAGTTTGTTCTTCTTCCGGCACATCTGGCAAATTTAATTTTGGTTCTTCATCATTTTGTTGTTGTTCATCAGATTCCACTTCATTGTTGTTTGTTTCATCTTCAGTGACATTAACATCTAGTGTGTTTGGTTCTTGAAAAACATCTTGGTTTTGATTAAGGAAGTTAATCAAATTGCTACCTCCAGTATCAATGCATCTAAGATTCACTTGTTCTTCAGGTTGATGTTGTTCATTTTCGGTTTTGGATTTCCGATCAACTCTACTTTTCCGTGTGACTTCTGCTTCAAAATCATTCACAAAAATTCCTTTGATAAAATTCCAAACTGAAAATTTACCTTGAGGAACATTAAATTGTGCTTTCAATATTTCTTCATAAAATTGAGCGGGAGTTCCTTCACAAACATTGTTTGTTCCGAGAATCTCATTCATTTCCATGTGTAAGTCATATTGTTCTTGAATGAATTCAATGATTTTAGCATATTTAATGTATGATGTGTATGGTGTTGATGTCATTGGATCCAATAATCTAAAATTAACCCATGCCATATTTTCAATTTCCGGGATGGTTAATTTAGTTAAATTGATGTTGTTTGTTGCTGCAATTCCTTTCCATTTACAAAAATTCGTGTCTAGACTCATTTCAACAACAAGATTTCGACGACGGAAAAATGCATCATGTGATGTCAATGATGGTGGACACCAATGTGGACTGTTATTGATGGTAATAACACATTTAGGTGTTGCTGCTGTACCTTTAATTCCAACTGCTGCATTGTCAGTTGAAGCCAGATTTGGTCGATAAGGCCCACTGGAAACTAGATTCAAATATTCAGTTGCCATTGCGACATTCAAGTCACTATTAATCAAAAATTCATCATAAACAATGACATCAGATGAAACAAAACCATCCCAAAACTCAGTATGTGGTGGTCTGTTATAGACATCTCTTGTTGTGAATCCTAATTTGTTAATAATCTTTTGTACGATTGTTGTTTTTCCAACTCCAGGTGGTCCAGCTAAATGAACTGAGAATGGAATTAATCGAGGTTTTTCAGCATGTTCTTTTTGCAATAAATTTCCCCAAGCTATGTTAACACGATTGAATAAGTTCAACATTTCAATTCTACGTTTTGAACTAACATGTTTGCACAAATCAAAACCTTCTTTCTGCACTTCAGCCAATTTTTGCATATAAAAGTCAGATTTGACTACTGATGGAATTTTCATCAAAGATAACACAGTTAGTGCACGCGCTTTCCATTCATCTGCGTGTAAACCAACTTTTTGTTCACTAGATCCATACCGTTCGATAAGAGCGGCACGTAAGGCACTTGGAAACATAGCAAATACACGTCCACTCATGTTAATGCAAGCATTAAGAGCACCAGCAATTAAGATCATGTTTGCACAATATTTCATAAGTACTTTTGCATCAACTTGCATAACACTAGCTAACAAGTAAACTATGCCTTCCATTGCATTCTTTGTTTGTTTGATACCAAAACATAACATTCCATATATACTTTGCCAATTAAGAACTCCCATTAAAGAGAGTAATAAACAAACACCGATAGCAATTATTTTGGGCAAATATTCAGTCTTCATTCTCTCATAATATTCAGAAATACATCCAAATATTTTGGTGACAAAAGCTGAGATAATTTTTTCAAAATAATCATCAATGATTCCTTTCATCCATGATAGAGCACCCGTAACTAATTGACATACTTTTCTCAAAGCTTCTTTTAGACAGGCGGTAAAATTGTGATAAGTCATTTTTATCATTTCAGTTATACATGGAAAGTTCAACCATTCCAACATACCTTCAAAAGTGTATTTGGGTGCATTGTCAGATTGAGCAAACCAGTATTCATATTTATGTTCAGGTAATCCTAATGAGATTGCTATCTTCTTAAGATTAACTGGTGAAACATTCAATGGTGTATAATCGAATTGGTTAGATATTGTTGACCAATCGATAAATTGTAATTGTCTAGCAAATGCGCTTTTACGCAACATTGCTTTAGCTTGTTTTTGTTGTTTTGCTGTTCCCAATACATGTAGGGGTTGAAAATTTGCGATCAATATTATCTTCTCCAGCCACATTTGATAACATTGAGCACAATGTCCTCTATTTTCAAAAATTGTACGACAAATACAACTCTTATGGATATCACTATTACAATATGTGACATCTTGAGGACAATGTTCAAAATTGGGTTTTCCATAATTCTTATTCATTCCACGTTTAGTTCGCATTTCATTAACAAATTCTAAATCCTCTTGTTTTTCAACAAGGAAGGTATTTTCGAATTGCAATAAATAACATACGCCACCATGTTTTAAATCTTCCGAATTTTTCTTAATGTAATCAATCAATACATTTCGATCAAAATTGATCAATTGGTATTGTTTGACAACTTTAAAATTTTTCCTGGCACATCGATCTTCGTGTCCAGTAAAACCATTACACTTGGGTAATTCATCCAAATCAGAGATAAGATTTTCCAAGTTTTCACATGGTTCATCTCCTCCGTTAACTGAGAATTTTCCAACTCTACGTAATTTTGTTCTTGCTCCCATGTTAACACCAGAATTTGCATTCAGGCCTAACAAGTTCATGATTGGTTTTTCAACCGGATTATTTTCTTCAACAGGAGTTGAAGTCTGTTTTTCAACAGGGTTTTCAGGCATTGGTTGTGATTGTGAACCTGATTGATTGTTAACCAATTGTTGTAACAATGGTTGAATTGTTGTTAATAAATTAACTAAAGACTGGTTTTCACCTTGGCCTTCTTCTTGATTTTGTGATTCGATTTGTGATTCACCTTCAGACATTTTCACGTCAAATAATTTTAATTAATGAATAAATTTATAGGGCAATACTTAAATAAATTTATTTGAATTAATTTTAAAAGATTTACAGGGCAATACTTTAATAAATCTTTAAATGAGCAGAAATACAGGGCAATACTTTAAATAGATCTGCTTGTGTTTAATTTGTGATATAGAAATACAGGGCAATACTTTAATATTTCTAATCTTTGAAATTTGTTCGCTTTCTTTCGTTCGCTTTTTGTTTGCTTTCGCTTTCTTTGTTTGCTTTCGCTTTCTTTTTCGCTTTTTGTTCGCTTTTTCTTTCGATTTCGTTTTTCTTCGCTTTCTTTCTTTCGTTTTGTTTAATTCGCTTTTTGGGCTTTCGCCTTGATAATTTTTGTTGAATTTGTTTAAAAGAGTTTGTTTTTTGCTTTCAAA

>Carpotroche-associated ilarvirus, movement protein

CGTATTACAATAGTTATTCTTCTTTGTTATATTACTCCATCTAACACTTTCTCTGCTTTTTTTTTTTAATGATACGGCGACCACCGAGATCTACACTCTTTCCCTACACGACGCTCTTCCGATCTATCTAATCTCCTCTCCTGAGAGAGAGATTAGGTACACATACATAAATGCATGCACGTCTATAAACACCATATAGGCATCGTTCGGGAGAAACATACGAACGGAATATAAACTCATCCAAATCAAAGGCACTGAGTGCACCCCTGAGGGCATTTGAAAGACGGACATAACAGTCCGATTTCAGCATCTGCTCCACAATACAGGCATTCGTCTCGAAGACTAAAGTCGTCGATTCGAGTATACGTTTGCAAGCCCCAAGAGACTTCGCAAACCAAACATCGACCCCCCGAGTGTGGTCACTTCTCGCAAGCGAGAAAGTTCTGCGGAGGCTTCATTGGTGGGAGTTTGGCAGTCTGCCACCACATGGAATGAACTTTAATACCGCTATTAGCGGGCATTGTAGTAGGAAAGGCCGCATCAATGGCCCAAACAAAAGCATGAGACCCATTAAGGTCAGACGCAGTGGTGCCAGCTGGCATAACCATTTCCAAACCACAGTATTTCCCATTAAGGAAACGATTTCGATTCGGAGCCTCAGGCTCCTTCAAAGCAGCCGAATCGAAAGCATTGTCAAAACCGGCATAACCATCGGATTTGGCAGTAAAGCCAATCAAAACTGAATACACTGCAGTGTTAGCATGTATCAGGTCTGGAAACTTCTTCAGTTCAGCTTCCAATGTGGTATAATACACAAGAGATGCGGAGGAACTCCCAAAGGAGTATCCAGACATTTTGTGCCACTCACCACTTGGGAAATTCGTCGGTAAATTGTATCCCAACGAAACTGGTATAGTGCCAATCATAGGCTGTGTAGCCTGCATACAACTATTACGCTGTCGATTCATAGCGTAATTGCGTGAACGATTAGTGGGTTTACCACGCCGCGGGGCTGAGCCCGCACACGGCGGTTGACATCTTCTGCCCATATTGGGCAACTTAGCTCCACCACAAAGAGCACAGAAGGCATTCCCTTGAGCATTCATGTTGTACGAAGAGAAAGAATTGAAAGTTTGAGACGCACGTCAGCACACACCGCGTGATGCTGCCCGGTCCTAGGACCGAATCCAACAAAACTTATAACGGTGTATTCGCCGAATTCCCGGCGTTCATCGCCTTCTCCTCAATCTCAACAACACTTGCCCCATTAGATTGAATTTTTACATTCGATTTGGGAGTACTTGAATCAACACCAGACCCTTCAGCCCTATGCTGAGTATAATCCACAATTACATCAGGTAAAGAACCTGGAAGGGGTGATAACAATTTAGCATCTGCTAACATGGCTTGCTGTGAATTATGGACCATACTGGCCATGAGTGAGTCTCTAACAGCAGCCGAAATGTCATCCTTGACCATGGATTCCTGTTTGAATTTCACAGTCATGGGAACATCTTTTTGGTAAGTCATTTTCATTCCAAAATCTTCTTCCCAAAAAGGTAACCAGCGTCCAGCGGAACAGTCGGCAGGTAACGTAGGAGCTATAATCTGATGGGTGACACAAAGTCCCCTACCATTTAAAGCATCTTTAACTCGTAACGACCTAGGCCAACCAGTGCGGATGAAAAACATCTCATTTAATGGTAAAACGCCAATGGGGATTTTCTCCAATGTCGCTTTATTCACTAAGTATGATGTGTCAACGCAACTCGTTGAGCGAAGAATTCTGGGAACATAACAGAAATAAATATTCCTGTGATCAACATTAAGTTTTCCCTTGATCTTAGCTTTCCAAGAATTGGAAATAAGACCAAATTCTTTGCTCAGTAAATCCCAAGCTTGAGCACCTGATTTGCTCAACTTGGCTTCGACTGGAACACAGCCTCGGAAACACGTCGAGGCTACAATGTTATCACGTAAGACACCTGAAATCTCCCCCATCAGGGAGTCCCAATCTTTACCTTCATAGGTGATCTTTTTATATGTGGTAGAAAGAGCCATCTGATGTTTGGTATTGATATCCTTCAGATGTGTAAAAAAAAATACACTTCCGGTATCAAGCACAGCGATTTTAGTGCTTGCACGAATTATTCGCACTTCTTCACTGCCTCACTCTCGATTTGGGGAAGAGGAGTGTGAACAATCTCTCTGGTCGTGATTTCGACTCAGGAATGTATTCTGGTATTCGTCGCCCGGAATCCACCGGTTACCGATGCTCAGTATACGTAAAGCTATGAAATTCGTCCTCAGTATCCGCCGAGTTAGCAAGGTAGAGGGAACAAAGTTCTGTCGCCAAGTTAACAAACTTCAGAACAATACGTATAGCTTGGTTGATCAGTGCTGCGAATAGATCGGAAGAGCACACGTCTGAACTCCAGTCACACTTGAATCTCGTATGCCGTCTTCTGCTTGAAAAAAAAAAAACGCCACTCCCGCGTATTCCGCCTTTTTCTTTCAAATCCCCATACGTGTTAATATGTATGTACATACCACTCCTACATTGTTA

>Carpotroche-associated ilarvirus, replicase protein 2a

TATTTCTATATATACTTACTTCATATATTCATTTTTTTTTTTTAATGATACGGCGACCACCGAGATCTACACTCTTTCCCTACACGACGCTCTTCCGATCTATTTTTCTCACGACCCGGATTAATCACTCTGTGAGTATATACCCGTTAGCCACTCCATGGATTCATGTGTTGCTTCTCTCGATTTGTTCCTTATTCCCAAGTTTGATGTTTCTGTTTATCAGAGTTTCTCTCTTGGTTATGAGAGTTGGTTGGAATGTGAAAAATTTCTATTTCGAGGGTTGCTGTTGCGCCATCTTGTTCCAGCATTGCGATTTATGTTGACGAAAGATACCATCATGGATAGGTTGAGCCTGTTCGTACCTGTCGTTTATGATGATGAAGAAGATGGTGCTGTTACCGTCGATGTTTCCGTCTCGACTGTCGTAGACGATTGTGATTCCAGGTTGGATTTTGATTCGCCTGATTTCGACGAAGATTTTTACCTGCAATCACTGGGAGCTTCGTCCCCTGCTGATTTGTTGAAGGAGGAGTTTCAGAAACCCTCATTCCTTGGTCATGAACATTGGGGTTCTGTGACCACTGATGAATCGATTGTTATTGATGATGAGATTATCGAGGATGTGCAAGATATTCATACCATCATGCCGATTGATTATCAACTCTTAAAAGATGAGGGTTGTGTTGAAGCGGATTGGTCAATGAATTTTGAGGCGGATAGTCCTCCTAAGCCAAAATTTAGACCATCCACTACCGATGTGCAGTGCGATCCCAATGTAATTCAGGCGGCCATCGATGATATCTTCCCATTTCATCATGAGATGGATGATCGATATTTCCAGACTTGGGTTGAGACTCAAGACATATCTCTTGAAGTCTCAAAGTGTTGGTTGGATGCTTCAAATTTTAAGGACTTTACTAAAGGTCAAAGCACATATGCCCAACCTCAATTCCAGTCTGGTGCCACTAGCAAACGAGTCAACACTCAACGTGAGACTCTGTTAGCAGTGAAGAAGAGGAATATGAATATACCCGAACTGCAGGCGACATTTGATTTAAACGCAGAGATTGAATGTTGTTATAACAGATTCAATCTCCATGTTTTTGACAAGTCGCGAGTTCGGAGATTACCACCATTATCAGGGACTGAAATAAAATTTTTCTCTGATTACATTGTGGGGAAAAATCCCCCTCTGAGTGAATATGTTGGTCCTTTGAACTTATGTTCGTTGGATAAATATATGCACATGGTGAAGACGATTGTCAAACCTGTTGAGGACAATTCGTTGAAATATGAGCGACCTCTTTGTGCCACGATTACTTATCATAAGAAAGGAATCGTGATGCAGTCATCTCCGCTTTTCTTAAGCGCGATGTCTAGGCTGTTCTTCTGTTTGAAATCGAAGATCACAGTCCCTTCTGGGAAATTCCATCAGCTGTTCACCCTCTCTGCAGCTGCATTTGATGCTGCTTCATGGTATAAGGAAGTTGACTTTTCAAAATTTGATAAATCTCAAGGTCAACTTCATCATGAAGTTCAGAAACTCATCTTCAAAGATTTGGGGCTTCCTAACGAATTCGTTGATATGTGGTTTACTTCACATGAAGTTTCGCATATAACGGATCGTGATACTGGAGTGGGTTTTTCCGTTGATTATCAACGAAGAACTGGTGATGCTAATACCTACTTGGGCAATACATTGGTTACTTTGATATGTTTGGCGCGAGTGTATGATTTATCCGATCCAAACATCACTTTTGTTATTGCCTCTGGAGATGATTCACTCATTGGTTCCACGATGGAGTTGCCGCGGAACAATGAAAGTTTATTCACCAGTCTTTTTAATTTTGAAGCCAAATTCCCTCATAATCAACCTTTCATATGTTCGAAATTCCTAATCAGTGTTGATTTAGAGGATGGAAGCCGCGAAGTTATTGCGGTACCAAATCCAGCCAAGTTGTTGATCCGCATGGGCAGAAGAGATTGTCAATACAATTCCCTTGCGGATTTGTTTACTTCTTGGTTAGATGTTGTGTACTATTTCCGCGACTCTGAGGTGTGCAGGAAGGTTGCTGATTTATGTGCTTATCGGCAAAAAAGGGGTCCTTCGGCATACCTGTTACCAGCATTGCTGAGTCTTCCGGTGTGTTTTTCTAATAGAGCTAAGTTTTTACGGTTATGTTATGATTTAACACCCGATGTTTGTCTGAAGCAAGTTAAACTTAAAGCTGATTCTAAGAGAAATTATTACCTTAAGAATCATGAAAACCGATCTTTTGGGAAACAAGATCGTAGACAACAAAATGGTCGTAGAGGCTGGTTCAATAAAGAGAATGAGAAGAGGTCTCTTTGTTATGAAAAGAAAAATGAGGATGATATTCCCTCAGTTCTTAATGAGCCTAATAATTACCGGTCATTTAGTAGCCGAAGCTCGGCCTTTGATGGTCGTGGTTCCGAGCGAAGCCAGCCCAGAACCACCAGCGAAGATGGTCGTAAAAATTCCAGACTTAAATATCGACCTAGAAATACTGGAGTTCACCAATCCTAGCACTGTTGTCAGTGTTATTTACCGCCGCGTGATCGGCGAAGTCAAGGAGAGTTGGTATGGTTTTCCAAGTTGGTCTTCAGCACGTTATTCGAAGACTTATGAGTACTTGAAGCGTGTGTCTAAGATGCAAGTTCATCTTAGTATTCCAGGTTCTGATTGGGCTTATACCCTATCATTGTCTGATGTGGTGTCTGGGTTTGGCTTACCCAAAATACCAATCCCTGAGTCATATCTCAGGTTACCATCAGTGAAGTTTTCACATGATGAAGTTCGTTAGTCTGATTCTCCGGAAATGCTCAGCAGTTCGGATTAACGAGTTTATGTTCCAATCGTTTGTTTCTCCCGATTGATGCCTATTGGTGTTCATAGATCGGAAGAGCGTCGTGTAGGGAAAGAGTGTAGATCTCGGTGGTCGCCGTATCATTAAAAAAAAAAAATGTACAATGTCATGTGCTACGCTTATTTACGTGAGCTTCATCACTTGAATCAGTATCAATCGTAGTGAATGTGAATATACACCAT

>Carpotroche-associated genomovirus, replication-associated protein

TCAGGAACCATGCCGTTCATATACATGTGAGTTCCTAAAGTGGTAACCGTTAGCCGAGTGCTGTTTGAGGCTGACCCACCACACAACCGATTTAGGGTTTCGCGTAAGCGAAAACCCTAACATCCCCCTTTTAGGGTTTAGGGGGTTTAGGGTAGTTCGCGAAGCGAACATCGCTGGTCCAGCGCGAGGACAGGATAAGAGTAATCGCCGAAGGCGAAACCGGATTCCGGTTTTTAGGGTTTAGGGGGTTTAGGGGGTTTAGGGGGTTTAGGGGGTTTAGGGGGTTTAGGGGGTTTAGGGGGTTTAGGGCTTACCCAATGAGCGCGCCCACACAGTCTTGCCAGTTCTCGTTGGACCCCACAAAATCAGGCTTCGGGGTCTTCCTCCCCACCGCTCTCCTCCGAGCTGGGCCTGATTGACCCATTGCACCAAGGCGTCGATCCGGTCCGTCTCAAACATGGGGGAGGTGTACTCCGGAGGGTCCTCCGCGAAGTGCCTCTCCACAAATCGTAGAATCGCCTCATGGTAAAGCACGTAATCCCGTGGAGCATTCTGACGTATGCAGTCGAGAAACGCTTCTTTACAGCTAGCAGAGAGAGCGTCTCCCCAAGCGCGGTCACGTCCATTTCCAGAAGCTCGATGTCCTCCAGGTTTCTCTCCAAATTCATAAATGATGTCGTCGTCCTTGCCGGCATAGTCGTAAGCCTTTGCAGGCGTCGAACGTACGCCTCGGACATTAGGGTGATAGCCGTGATAGTCGAACAGTCGCTCATTCCGTGTTGAACAGCGATCCGCTGTGCCGACAAAAGCATGGAAGTGAGTTCCTCCATCCTCATGAAGTTCTCGGCCAGATCGCCCTTGATGTGGTTTTCGGTGATTCCAAGAATTACCGCAAGGAAAAGTTGACATTTGAGGTAGTGGATTTCAAGAGTGCTTACCATGC

>Carpotroche-associated ilarvirus, replicase protein

CCGAGATCTACACTCTTTCCCTACACGACGCTCTTCCGATCTGCATCTCCTTAGAGGCATCTTATCTCCTCTCCTGAGAGAGAGATAAGGAACTTATGTGACTTGCACATAATCTTCTATAAACACCTTAAATAGGCATCCACTGGGAGAAACATACAGTGGGTATATAGAAATAACTCAACAACGTCTGAGACTGTCCTGGGACTATTGAGTTACATAGTACTAATTCGATATCGAGAAAAATCAGTTAGAGTTTTTGACCATCTAATGGCCTCACTCACCTGATCGTCTCTATCATCGGCATAGACTGAATAGTATCTTAAACTCTTCCTATGACGTGTCATTCCCACAAGATTGTGAGAATTACCGGCATCGGGCATTTTACCAGAGTAAAGTAAATTAGTCGTTTTCGACAACCTAACAAGGATGACATTATCAAATGTCTTTCCCTGAGCTTCATGAGTGGTCATAACATTCATGGGATTAATACCGGGATGCTTCAACATTGCACACTTATCAGCTTGGGTGTGCATTATGTAAAGAGTGTCTTTCTCTGCTGGGATCTCATAACCACTATTAATGTTGATCAGAGATAATGAAGACTCTCTAACATTATGTGATGAAATCTTCGTGTTATAAAATTTCTCAGATAGCACGGCAGTAACATCCAACGGACACCTGTATGTGATTGTCTTAGGCTTCAACCTTCCCACTATGGATGCACAACGCATTCGGAAAGTCGGTATCCTAGATACAAAGGGTATCTGCTTAGTATCTCCAAAGGCAATGACCTCTTTAACTTGAGCTAAAGTGGCAGCAGCATATATGACCCCACTATGTACAAGGAAACATTCATCTACTAATAATCGTTCAGCAGAATACCACTTGCTGAGCATTAAGTAGGAATCCAATGTGCGAACCCGGTACAGTAAAGCCGCTGGAATGACTCCTGATGCTTTTGCATCTTCAGCAGTTTCACGGTTGGCGCTAAGAAGTAAATCAACTTCGGGTTTCGCCTGTTTGAGCAAAGATGTGGTTTTACCACATCCAGCAACACCATCCTCAATCAAGACTGTAAATTTAGCATCCATCGTCAGAGCTTTATTCAAGCCTGGCATCAGTCTCGATCGGCTGTCGAATATGCAGGTAGAATCCGCAAAACATACTGCATACTTCGAGATATCGGTCCAAGAAGCTGGTGTCAGATCAGAATGATCTTTATTCCAGACAATTCGTTTGAACCCATCGACGGTCATGACTCTCTCATATGGTTGAAACTCCACTCCCGGAGCACGATACCATTGCCGTCGGTGCACGTCGAGAACACTCAACGTCTGATTGCCCCCGTACAAAGAAGCATACGAATTCATCACGCGCCAACCAGTATAGTCACCACAAATTCTCAAGTTTCTAGTGGCGACTACTTCCAACTCATTGAAGTACGCAATTGCTTCCTTTATTGAGTCGGCATATGGATTGATATGGGGCTTATCACTATCCATGGACATGGGTTTAACGACTACCTCGGCAGTTTTGTTGCTGATTAACCTAGAAACCAAAGGTTCATCGAGTAGTGGCAATAACCCAGATTTTTTCTTTTCATCGTCTCCTTCCAAGATATCTGCAATCTTGACAAGGGCATTTTCCAACTTCCTCTTTCTTTCTTCTTCAACAATTGATGCCGCCAGCATCTCTTTTCTTTGTTCGGCATACCCAGATTGAAGATCAGAAAACCATTGATCAACATCTAAAGTTTTACTCAAATTGTTGAATTCAGAGTGGCAAACACAAACTTCACTCATAAGAAAGAAACTCGGTACTGATTGAATGACAGCATCGTCCTGATCCCATAGTTCCATACCAAGAGCAGCCTTAACTGCATCAAGTAGCTCGGAACTGAGACTATGACGGTAAATGAAAGTCGAATTTTCATCAACTTTCTTTTTCAATGGGGTCCCAAACAGTGCTTTGCTCACTAAAGGGAACCGTCCAGTCAATTTGTCATTCTCCTCGGCGATGGAAGTTCCACGTGTTTTCACAACGTTCATCATCGTCGGTATTTCATCGTACCTAGCCTTGGCATATGCAACAAACGTAACCGCCAAAGGCACATAATCTTCAATATCAATGGGCTTTCCAGTATGCATCGTGACCCCATTGATTATGGTATGATTGGAAGCGGAAGAAATCATCGTCGCAACAGACTTAACCAATTCTGCAATCGGCGTGTCTTTCTTGTATTGACGGAAACTAACCTCGGAAACGCGTCTAACGAAGTCAGTATCCAACATAACGTATTTCACTACGTATGAGGACCTGACCATCATAGGCACCGCCAATCGCACCAAGGTCTTCTTGCGAATGCTATGCAACCAAGCACATGAAACATCCTGCAAGGTATTCATAGACAGTCTGTCGGAATTATCGCCAGCTAGAGTGATTTCAACGATAAAGACCCCATTGAGGTCAATAGTACGTTCAACTCTATAGGCGGCCTTCCCCTTAATTATGACTTGATTGCATGTCATATATTGCATCAGAATATCATAATTATGAGTATATGATAGTCCGGGAGCATCGACAAAGTGGAATGATATGGTCTTCTCACCAGTGTCGAGTTCTTCAATTTCCCAAATGACGTTGAGATACGGAATAAAACCCTGCGTCGCAACTAACATCATTGGGTCCATCATCACACTACCAATGAGTTTGATGACACCTCGCCTATGCATATGCTTCACTAATGAAACAATCGGTATATCTGACATCGAATGTATCGCCATTGCATAGGGGGCTTCAACATTGCAAGTTTGAAATTTGTTTTGACAATAATTCAAATTCAACTCGCGGTTGGGATTTTTTTCCAATGCTCCAGCTACACTGATAAACCTATCAGTGAAGCGAGCTCCATCTCTAACATCCAATATTGGGCAACAGCTGTGAACATTTGTGCGACCCATCTTCGCATGAGTGCAAAAGTTTCCTCCAATATCGATGATAGGCGTCTCTGATGTTTGAAATCGCGAATATATGTAATCAGTCTCACACACTCTATGCGCTGCTGCGAAAGAATGAGATGATGCTGAAGAATTCCGAAACTGCAATTCTCTTCCAGGAAAAGATTTTCGCAATACGCTCTGCTCCTCAGGAGTGAGGGAAAAAGAAACATTCAGAGGTACAATTGGTGAAGAGTCGATATGCCTATTCACCACCTTAACAGCCGCGTCTGCTACGAATCTACCAACCGAAGTAGACTCATCCGCTGCTCTTCGACGGATGACATCGGATATGATATCATCCATACGAATACAAGACTCGGCATGTTGAACGACAGACTCCATTGCTAACGGGTATATACTCACAGAGTGATTAATCCGGGTCGTGAGAAAAAGATCGGAAGAGCGTCGTGTAGGGAAAGAGTGTAGATCTCGGTGGTCGCCGTATCATTAAAAAAAAAAAAAATAGAGAGCGATTGGTGGAATACAATGAAAAAAAAAAAGAAACAAGTGGTCACTAGGATACATAAAGACGGCTACGCTGCGACAGTAGTCACAATACTGTCCAGTCC

>NODE_9936 (unclassified Picornavirales), polyprotein

TAATGATACGGCGACCACCGAGATCTACACTCTTTCCCTACACGACGCTCTTCCGATCTATCAGGAACAGCATTACTATACTTACCTATTTCACCATATTTGAGACCATCAAACACAGGATATGGTCCTGTGTTGACTTCAGAGGGAACCAATGCTTGGTTTCCATCAACACTAACTTTTCAACCAACTTATAGTGTTCCTTTGATTTCCTATGTTTCAATGCCATTCAATTTTTGGGGTGGATCGTTAAAATATAGATTTGAATTCATCACAAATGCTTTTGTGACAGCCAAAATAGCATGTTCCATAATTTATGGTACATTGAGTCCAGACACGGTCACAAATGGAATTGAACCAACGAGTAGTTTGAGTTATTTTTTTGAAGTTAATGCAGATAATAAGATTTTTGAAGTTGAAGTTCCATATGTAGCAGATACACCATGGAAGAGGGTTTTGAGATCCCAAGTGAATGGAGGGACAAATTCAAACTATGATGATATGACAGCCTATGAATATTCAATAGGCCAAGTAGCATTGTATGTTTTGAATCCATTATCAGTACCATCAGGATTGCCAACTTCATATCAAGTAAACGTATTTCTTGCAGGAGGCCCAGATTATCGTGTGAATTTTGTTTCAAGAGCGAATACAGCCTGGTTTCCAATAGCCCAGGGCATAGACCCAAATCCAGGAACCGATTTATCAACATTGGGAATGGGTATTATGAAATCAGATATGAATTGTATGTCAGAGGTGTACACTAGTATTCGAGACTTATTGAAAAGATATTCTCATGTGTTGACTCAAACCATGCTCGTACAAGGAACGGGGGGTTTTGATGCAGCATATTATTCTCGTTCAATAAGTATTCCAATATCAACACTAATAGCACCATATTATGCATCAACACCATCAACAATAGCTTCCACAGTTCAAAATTGGTTTATGGCTTTATATAGATTGAACCGTGGATCATTGAGATTCAAAATTATGTATACGATTGAGGAAGATATTGGTGATATTGATGCATTGTATCGTGCTATATCTGTTGATTATTATCCAGACAATATGAACTTCAAAACAGATGTTTCTGGTCGAAATCTATGTATAGGCGCATCCGATGAATCAATTGGAGCAACGACAGCAAATCCAGAAAATACGTATCAATTATTAAATAACACAACACATTATGGACCAAGAGAGATAGCAAATGATACTGGTTTTTATAGCGAGATAGAAGTCCCACATGTATATCCAAATAGAACTAGACCAACACCATTGTCTGGTAATGAGGGAATTCAAGACTTGGCTATGAATTCAGTACCTGCAAATAGAGCTGTCACATTTGTCAATTCGACGATGCGATATGGATCATTGATGATCAATGCACCACCAGCGCCATTAGACTATAATTTGAAGATACGATTATTTATGGCAATGGGTGATGATTTCAGAGCGGGATGTCTTCTCGCAACACCAACCATATCATACGGAGCATTGAAAGGTGTGCCAGCAACAGCAGCATCATATGCTATACCACCAGATTACTATAATTAGTAGAGAAAGAAACTCTAAATAGTGAAATTGATTTTAGCACACTATAGGTGTGATAGGGCCTATTTCATGGCTTATATATAATAAAATTAGGAAAGATCGGAAGAGCGTCGTGTAGG

>NODE_15442 (Rhabdoviridae), N protein

ACGCTATTGCGATCTTTTAGCTCGATCGCGGCGGATTTCGCTGACAATTCGCTTTTTTTTTTTTAATGATACGGCGACCACCGAGATCTACACTCTTTCCCTACACGACGCTCTTCCGATCTCGGAGGAGGTTCTTGCCCTGTAGATGTTTGGGGATCTTGGGATCTATTCGCCACTGGTGTTGTAGTTTGGTTGGGAATTCTGGGGACATTACCTGTCATCATAGCCCTGTATCTGTCAACTGGGCCTGCACCTTCTTTTATTGCTTTGCTCCTTCTGTAAAGGTTCAATACTATTTCTGCAAACTCCTCTGCGTCTTGCTTCAAAGAAGTTTGTCCAACTAATACAGCCATGTTTTCAGGATCCGAATAAGTCGCTCCTGATGTCAGTCCCAATCTCTTATGAAGGATTACCAACCCATATAACAATTCTTTATTCCTCTTATTCTGAAGAGGAAGAAAGAAACCCTCATTGATCATTCTGGCATATTTCCAGGTGAAATCCCTTTGTTCTCTGGGTTGTTTGGAAGGAGCATCAAGCTGAGTAGCAATATCGTATATCATCTGTACTGCTTCAGATGTTTCCTCCACTGAAAGATACGTAAGCAATAAAGATGGAGATATCCCCTTCATTTTGTGCATCAATTGGACAGTCATAGGGTATGCATGCATTCCTGGATATGAGAGGTGTTGACCCACAAAATAAGTGAACATATGATGAGGTGGAGTGTTCCTATTGAGACGCTTATCTGATGCTCCGGCACATAACACTATATAATATGTTATAGAAGTGTATGTACTGAATGCCACCTTCACCATTTGAGCATATGTGGCAGATGGAGGGAAGTCAGTCACTATATTGCTTGGAGTACCATAGAACACTCCAAATTGTTGCTTCACTTGACCAATTGCTCTCCTCCAATTTTCTGGTTCCTTCACTATGAGTCTCAGGAAAGATGATGCAAGGTATGCCACGGACAGAGCATCTTCCTCAGTTTCCTTTAATCTCTCGGATATTCTCGTCTGGTCCATGGGTTCTTCTTCTTCTTCATCAGGATTTTCCCCGCCCTCCGTCCTTCTGTTCAGGGCTGAAGTCAAGAAACTTGCAGATTGAAGAATACTTGAAGGCTTTGGAATCCTTTTTGTGTTATCAGGATTCCCTTGCTCTACTTGCCAGTCATTTGTTAGTTCTGGGAGATAAAAGACAGAATTTCTATCTCTGCCTACCAGAGATAATGCTGTCGAGCACAGCTGATACAATGTAGATTCAGTCACTTCCCCTCAGATCGGAAGAGCACACGTCTGAACTCCAGTCACTTAGG

>NODE_18845 (Rhabdoviridae), G protein

TATTACTGCTCTGGGTCACTCTGTCTTTCTTGATATCTGTCTACATCATCAGTGAGTACATGAACTTGAACCTCTTTTTTTTTTTTTAATGATACGGCGACCACCGAGATCTACACTCTTTCCCTACACGACGCTCTTCCGATCTGGGATGGAGAGATCCTTTAAGTCAAGATGAGCATATATACTATGTCTCTTCTTGTAGGAGAGTATCTAATTGGGTGATGGAATATCCTCTTTTATTTTGTCCAGGGAGTAGAGGACTTTATATCAAAGATCAGTCTGGAGAGAAGTCATTATGGAATCCTCAAAATACATATTTTATCAAGGATTCAGTATGTACAGATTTTCCGATAAGCAATATCAGCTTAAGGATTGCAAGAAATGAGTCTGTAATGATAGATTTCTGGGGAAAGAAATTGATTATTCATCCTCCTTATGGATATGAACATGATTGGGAGGATAGTGAGCTCCATATTTTCAGATCCTCAAAATGGGTACCTTATCTAAAAGGGGTGGATGTTAATAGTACAGATGAATTCAAAGTATTCCTTGATGGATTCAGGAATTATTCTGAAGATAGAAGTCACCAAGTGACAATAAAGTCTGAAAGTGCAAAGAATATAATCCTTGAATTCTTTGTAACTGTATCAACTATGATCATCTCGGGCTTTACTGGAGGAATTAAATGGATTAAAGACACCTTATTCTCATTGGGGTTTGTACTGAAACTTGTGATGTTCATCGGAATAACAGTTGGGATCGGAACTGTTATAAGAGCAGTTGCTGTATGGAGAAGTGATAAGCGAGTGGTTATAGAGAGAGAAGAGATAGAAGACACTCCTATGGTCGTGACTCACTCTCGTTTCTCAGGAGGAGAAGGAAGCGCACTGATTAGAAGGAAATGAATTATCTTGAATGTTGTATATAGTTAAATAAATCAGTGTGATTTTATGATTGTATTATATGTTTATGTTCCTGTTTTATTATGCTTTAAATGTTGTAATATTAGATCGTAACAATACTATTGATGATGTAACATAAACATGTCATTTTATTGTTATTCTAATATATCATTATATCGTGATTCTATATATATTAATGTTCATCCCAGATCGGAAGAGCGTCGTGTAGGGAAAGAGTGTAGATCTCGGTGGTC

>NODE_23163 (Mitoviridae), RNA-dependent RNA polymerase

TCTTCGCTCCTTTTCTTTTGATGGAATGAGCTTGGCTTCTTCGGATGCTAATGGCCACAGCTCTATTCTATTCTTTTTCTTTAATAAAAAAGGGAATTGGCTGCCTTCATTCATTCCTACCCCATCCTTAGGTCAATAAACCTAATCCCGAGCAGCCCTTTCTTTCAAGCTTCCGTGAGTCAGCTCGGTAGCTAGTCTACATTATCTTCCCTTAAGTCAGAGGGGTTTTGGATTCTTTTCTTCTCGTATACTCGGGAATGAAGCCTTCCCTTTCGCTGCTAACCGGAGTTGAGCTGGCTACTAAAGAAATTGCGTCAGTGATGGTTCCTTCATCAAAGAAAATATAGGAAAAAGGGCCTATCATATAAAAAGGGCTTTGAGAAAGAGGCCATCTATCGGTAGCAGACTTGAGATCAAAACGGAAAGAGTGTTTGCTTGGGACCTGTATAAAGGCTTCACCTGATCAAAAGTTCCATCCATTTGGATCCTTGCGGCGAACCAAATGTGGATCGGTCGGAGAAGTCTTTGGTTGATAGAATTCCCCATAGCTTCGATACGGCGCTTGCCAGCTCCCTCTAAAGTACAACCGAGTCGATCCGGTTCCGGGGGAATACTGAGATCATCACATGATGGCAAGAAAGGACCTATACGCCCTTCAAACCAATCAAGATCTTCTGGTGTGAAGGTTTTCTTCTTCACGTCAAAAGCGTAAGGCCCTCCCTTTATTTAGATTTAGGGCAACCTTGTTCAAATTGTTGACCAAGAACAAAATTCATAAGAAAGTGAAATGATCCCAACTCACGCCTTTTCCTTGATCAAATAGTGTAAGGGGAAAAAAAGCCTCTTCTTTCCCTTTCTCTCTTTTGAATTTCTTCCTGCACTGCTATAATATTAAGATTAGTTAGAGCAGCAGAATCTTAAAAAAGAAAATTGTGATATAAAGAAGAGGAAAAATGCCCATACTTCTATATAGTACGAAGCACTCGTAGCACTTGCTAAGAGTTA

>NODE_23836 (Rhabdoviridae), L protein

TTTATAACTTAATGTAGGATATCTTTTTTCTTCAGTTTTTTTTTTTTTTTAATGATACGGCGACCACCGAGATCTACACTCTTTCCCTACACGACGCTCTTCCGATCTGGAAGGAGGAGGGTGAAGACTGCAGAAGAGGAAGAAGTTACAATTGATAGTCTGATATACAATATGCAGTATTTTCCAAGGATCCTCGGAGGATATGGTATAGGATTATTACCTGAATGGGCCATGAGAGGTTTCCCTGGCCCCTTGACACTCTCAATTTCTTGGATTTATGGGATAGTAAAGAAGATAAATAGAAGTAATGGTTACAGGGAGAGATTGTTAAGAAGTATATCCATGAGTAAAAAAACAGGTGAGACGGAATATTTGCATCTGATAAGTGATCCTGTTGCTGTGAATCATGATCTTCCACAACACGGTCTTGCAAAGCTCAGAGAGGAGTCTGAAAAGGCATTAGTTTCTTCAACTTATGTGGTAAATGTAGAGTTTAAAGAACTTGCAGCTGTATGTTATTCTGAGGAAGCAAGAGAGTTAATTCAATCCTTAACATCATCCGATGTCTTAGAGCCAAAATTCCTCCATGAGATGTTCTCAGCAACATTATATGGTTATTTTAACTCTATTGTTGGAAGGATAGATAAGTCTGCTACAATCACCAGATTAAGCAAGAAACTTAATGTGATGGAGTCCATACGACTTTCAGAAATATCTTATATCATCACTACATCGGTGAGACACACTTGCAAGTATGATATGGTCCCTACCACTTGTGCTACTACAACCGCAACAATGTTCAGAGACATAGGATGGGGAAAACACATATCAGGGGTCACGGTCCCAGATCGGAAGAGCGTCGTGTAGGGAAAGAGTGTAGATCTCGGTGGTCGCCGTATCATTAAAAAAAAAAAAGCTACTACCCGAGAGCGGAAGAGCGTCGTGAAGGGAAAGAGTGTAGATCTCGGTGGTCGCCGTATCTT

>NODE_33142 (Botourmiaviridae), RNA-dependent RNA polymerase

TTGTGTGTTACTCCTACTTCGTTTTCACTTCGCTTTTATGTTCTTTTTTTTTTTTAATGATACGGCGACCACCGAGATCTACACTCTTTCCCTACACGACGCTCTTCCGATCTGTAAATTCTCGGCGGACGCGATACACTTGAGACCGCTTCACAAAGCGATCTATGATAGACTGTCGCGCGAGAAGTGGCTTTGCCGCGGTGACTTTACAACTGACGTTCTTCAGCGTGCTGGTTTTTCTTTTGTTCAGGGTGAAACACTGACTTCGGGGGATTACAAGAGCGCCACCGACAACCTTTCTATAGAGGTTGCGGAGGCCATTCTTGACGAATTGCTCAGGTCCACGGTCTCTGTGCCGGGATCGATGAAAGCATACGCCATGAAGATCTTGCGTCCTACGTTGTACAGTCTTGAACATGGTATTTCTGAATTTTCTCCCTCGCGAGGTCAAATGATGGGGTCCTTTTTGTCCTTCCCTCTGTTATGTCTGCAGAATAGAATCGCTTTCTTGTATGCAGGCCACACAGTCGGGATTGACAATTCGGAATTCCCATGTTTGATCAACGGCGATGACATACTTTTCCGCTCCGGTCCGCACTTCAGTGCGCACTGGATGGAGACTGTGAGTCAATTGTCGTTGGAGGTGGAGAGGACAGATCGGAAGAGCGTCGTGTAGGGAAAGAGTGTAGATCTCGGTGGGCGCCGGAGCATTAAAAAAAACAAGAGCGGGGCAGATCGACGGACGACTGTGACATACATAAAGAATACA

>NODE_39152 (Rhabdoviridae), N protein

GCATACGAGATACATCGGTGACTGGAGTTCAGACGTGTGCTCTTCCGATCTGGGCGGAGTGTTCCTACTAAGATTCTTGTCTGATGCTCCAGCACATAACACTATATAGTATGTCATAGAAGTATATGTGCTGAATGCCACTTTCACCATTTGAGCATAGGTAGCAGATGGGGGGAAGTCTGTCACTATATTACTAGGAGTACCATAGAAGACTCCAAATTGTTGCTTCACTTGACCTATTGCTCTCCTCCAGTTTTCTGGTTCCTTTACTATGAGTCTCAAGAAAGAAGATGCAATGTATGCCACAGATAGAGCATCTTCTTCAGTCTCCTTCAATCTCTCAGAGATTCTTGTCTGGTCTATAGCTTCTTCTTCTTCCTCTTCAGGATTTTCTCCTCCTTCTGTTCTTCTGTTCAGAGCAGAAGTCAAGAAACTTGCAGATTGAAGTATACTTGATGGCTTTGGGATCCTCTTTGTGGTGTCGGGATTCCCTTGTTCTACCTGCCAGTCGTTTGTTAGTTCTGGGAGATAAAACACTGGATTTCTATCTCTTCCTACCAGAGATAATGCTGTTGAACACAGTTGATACAGTGTGGCTTCAGTAACTTCCCCTCTTTCAAGATTTCCTATCCATGCCAGATCGGAAGAGCGTCGTGTAGGGAAAGAGTGTAGATCTCGGTGGTCGC

>NODE_39399 (Mitoviridae), RNA-dependent RNA polymerase

GTCAAGGTGAATACAGATCAAGATGAATAAGTACCCATTGGTTGTCCAGTATTATATTTAATACTGTTACCCTCCGGTGTACTAAAATATCTTGACTGCAAGATAGATTGTCAAGATTGAGCATTTAATAACGAGAGTTCTATTACAGGGTTCTAAATATCAGATCGGATCAGCCAATCCATAACGGGATCGGAAGAGTCCGATCCGATCCGTCCAAATTTGTATATTGAAGAAGAGTAATAGAAATTGGCTGACCCGGCCATATATTATCCCTCGAAAACGCCGACAAATCAGACAAGACACCGACAAATCATCCCTCAAAAACCAGTGTCTTCTTTGCAACCATTGACCCATCATCCTCTCGATCACCTGAGCTCTCAAAGGACTCTTTTTCCATGGCAGTTAGAATAGTTTCCTCCTGCACAGTCGCTATCTCCCGAAGCATCCACACGAGACAAATCTACAACTAATCCAAGACAACAAGCAGATCCACACTCATAACAAATCTACATCAAAAGGAAGATCTTGGCGCAACATAAGAGGTCTGAAGAAACACCCCAACAGAAACAAAAATACAGCCTTTATGAATCTATAGTAGATCTAGATATCCCATCATAAAAGGTAAGGAAAGCCACCATAGATCGGAAGAGCGTCGTGTAGGGAAAGAGTGTAGATCTCGGTGG

>NODE_41237 (Rhabdoviridae), L protein

GCCAGACTATGTTTATCTTTTTTTTTTTTAATGATACGGCGACCACCGAGATCTACACTCTTTCCCTACACGACGCTCTTCCGATCTGGGATGGAAATGGTACATCATGAATGATGACATCAATACAACATGCTTGGAGAAGATATAAGGGGGAATATGAGAGAATTCTTTGGAGCATAATGATTTGATACTGGTCCCCAGGGTTGACATCATGGAATGGATAACCATTGCAGCATCATCAGAAAATGGGAAGGTTCTAGAAGCCGTCTTCAAACTACTCCTAAGAGTTCTTCCTTTATACATCATGATTTTATTGTACATGAATAATTCACTAGACACCCATGTCTCATTTAGTTTTAATGGAAGACCCACCTGTTGGAAAAAAGAATCCAGATCATGCACAATACAATTCATTCTTCTTCTTAATTCCAACTTCCCTGCTGTTGTGATACTTCCTCTGGCATCCAGATCTTCAGTATCCAAAGTAATGACTAACACCTGATTGTCTCCCCCTCCAATCAATTGAGATGTAGCGTCGTGAGATCTCATTATAAACTCAATCATACATACAGTCATGATCGTCCATCCTTTTTGTCTTAATCCATCTTTACCTGAATGATCACCTATCCATGACCATGGCGATTCAGTGATAAAAGAGTCCCCT

>NODE_41497 (Botourmiaviridae), RNA dependent RNA polymerase

CATCCTCTACACCAGCATCCATCCACATCGACACTGCGTTAAACCTCCTTCGCTTACAGCCCCATTCAAAAAGGGTGGAATTTATCTCGCCAAACTGGTCGGAAACCAGCGTCTTTTCTTTGTTGACGATTAGTCCAACCTCAGATCCCTGAAGGACTATTTCACCGCGAAGATTAGTGGTCTTGCGCACCTCACGCAATAACAAGTCATCCCCATTAACCAAAAGGGGATGGCTGCTCCATTCTTTAAAACTAATCTCCTTCCTGTTTAGCATAGCTGTGAGCGCCATATCAACTACGGTCTTGTTGATGATGCACAAAAGTGGAAAAGACAAAACGGACCCCATGGGCTGTCCGCTAAATGTCTCCCTCCCATCAATTCGAAGGTTAGCAAGCACTTGCAATGCCTTATGTTCATCACTCGAAATGTGGTCAGCCATCTCCTCCAACACCTCAACTGCAACCCTGACGTAAGCCGACTTGATGTTATCTGTCGCCGACGAGTAATCAAAACTCAGTAGGGAAGCGCCTGTGAGACGCTGAACGTGCCGATCGGCTGGGTCTCCGACTAGCAACCAGATCGGAAGAGCACACGTCTGAACTCCAGTCACCTTGTAATCTCGTATGCCGTCTTCTGCTTGAAAAAAAAAATGAAATTAATTA

>NODE_45025 (unclassified RNA viruses ShiM-2016), RNA dependent RNA polymerase

TGTATCACTACCCCCTCCACTCTCACTTTCTTTTTTTTTTTTTTTAATGATCCGGCGACCACCGAGATCTACACTCTTTCCCTACACGACGCTCTTCCGATCTCGCCAGCTTCCTCTGCCCGGGGGTGACATGGAGTGTCCGAGTAGTCGGTAACCCCAGTCCACCCATATCCCTGGGTAGGAACCAGGACATGTTTGATGGCACTTTTCCTAGCACATCATGATTCAATGTTAGGAATCGTGTCATCAAGCGGTCCCTTTGCTCCCATCCCCAGTCGCGTATAAGGTCCTCGGCCATCCCGGCCAATGAGCGACCCACCGACGCATAGTTGGTCTCGTCCTTGTATAGGGGACGAAGATCCTGAACTACGGTGGTCTGAACGCGCACCTGTCCATAGAGCAAGCTGGTCTTCAAATGCGGAACGACGTCGAAACGATACGGTGCGGTACCGAACTCAAGTGGGAGGGTATCCCGGAGTCGGTGCACCTGACTGTTGAGAATGAGAATCTTCGGATGTGTGTAGTTCTTCCCAAGAGAGAAGCGCAGCCCACACCATTGAGATCGGAAGAGCACACGTCTGAACTCCAGTCACTTAGGCATCTCGTATGCCGTCTTCTGCTTGAAAAAAAAA

>NODE_47371 (Mitoviridae), putative replicase

CGCTCATCCGATCTCGCCCACCAAGGGACAAAGGAGCGAGAGCATGAATTTGCTCAAACAATCTCCCTACTTCCCTTTGGATGAATGATAGGATTATCATTACCGTATCCACTCGGTATCGTGCTACAGGAGCGGTTCCCATTTGGAAAAATGAGGCCACTTCCGCAGCATTAGTGGGAACATTCACTGCTAAAATTAGCAGCCGGATTCTTGCTGAGAGTTTTCCGATCGGTTTATTCAACCAACTTCGGACCCTCCATCCAGCACCCATAGCCTGCAGAGCAGTAGCAAAAGGAGTGCCTGTCGATTTGACAAGCATAGCAAACGCACCAATATTTCTGGTTGCAGCGTAAAATTGCTTAAACGCCACAGGAGAAACATCGATACCGTTCACTATAGTCCGTTTTGCGAACTCCAGTGCGGTTCCGGTATGAGAAAGGAGGGACTTAGAGGTGTTAACCCCTACTCCCAGGGAGTCCATGATCGTTAGATATTGGAGTGCTACTCTTTTATCTCCGAGATCGGAAGAGCACACGTCTGAACTCCAGTCACTTAGGCATCTCGTATGCCGTCTTCTGCTTGAAAAAAAAAAAACACACCTATATGCATTGGTGT

>NODE_48513 (Rhabdoviridae), N protein

ATACGAGATTACAAGGTGACTGGAGTTCAGACGTGTGCTCTTCCGATCTTCTTCCTCTTCAGAATAAGAGGAATAAAGAATTGTTATATGGGTTAGTAATTCTTCACAAAAGATTGGGGCTGACATCAGGTGCGACTTATTCGGATCCTGAAAACATGGCTGTGTTAGTGGGACAGACATCATTAAAGCAAGATGCAGAGGAGTTTGCAGAGATAGTGTTAAACCTTTACAGAAGGAGCAAAGCAATAAAAGAAGGTGCAGGACCAGTTGATAGGTATAGGGCTATGATGGCAGGTAATGCCCCCAGAATTCCAAACCAGACTGCAACACCAGGAGCAGGCAGAACTCAGGACCCCCAGACATCCACAGAACAAGAACCCCCTCCTCCTAAAAGAACAAGAACTGAACAGGGTGGAGGGAAAAGAGCAGATGCAGAAGCTGTGACGAATATGATGTATGACTGACTTTAAGGTTGTTTAATATGTTTTTATATTATAATGTGGGAAAGTATTTAAAAGATCGGAAGAGCGTCGTGTAGGGAAAGAGTGTAGATCTCGGTGGTCGCCGAATCATTAAAAAAAAATCAAAGAAAAAACCCTCTCCATCC

>NODE_48606 (Botourmiaviridae), RNA dependent RNA polymerase

CCTCTTTCCCTACCCGACGCTCTTCCGATCCCGGCGGTACACTCATCTGCGAGTGGCCCCTCACACCGTCGGCCCCGCGACGCTCATAAGCAGGGGACCCATGGCAGCTCTTAAAGCGCCAATCACAAGCCGAGTCGGCTGCGAAAGACTCCTAGGGCGAGCAACTTACCCTCGTACCTATGACCCTCATAGTCCTTCAGGAAAGGACTAGAGGAGACTACCGGTTGTTCTCTTTGAACCCTCCACCCTCTATAAGAGAGGGGAGAACGAAGAGGGATCTTCCGGTAGCGGTAAGACCTCCTTATGACCCCGATGGCAGGGTCATATCTCTTGCGGTCGCCGACAACTCTCCCGTGGGAGAAGATGTGGGCAACCAACGCTGCCGCCTCATCCGGGTCGGGGGTCTCCCCCCGTCGGATCTCGATCATCGGGTCGGTTTCAACCTTAGGAAGAGGAAGTTCTGAGATCGGAAGAGCGTCGTGTAGGGAAAGAGTGTAGATCTCGGTGGTCGCCGTATCATTAAAAAAAAAATTAGAAAAAAAGAGATCGGTGTGTAGAAAGTATGTCTATAACGATGATTGCAATGCGATGAAACAGACGAAAGAGA

>NODE_50646 (Rhabdoviridae), L protein

CCACCGAGATCTACACTCTTTCCCTACACGACGCTCTTCCGATCTATGGCAAGATGCTAGACGAACAGTTCTCAAATGGTTATCTATAGGAATCTCTCCTATGAGACCCTTTTTGACAAGAATTAGTGATAATGGAATGGCAATGAAAGATCTGATCATAGGATTATATGCTAAGGAGAGAGAGTTGAAATTACTGCCTCGGTTCTTTGCTCTTATGTCATTCCAGTTGAGGCTGTATTTTGTGTCGACAGAGCAGTTGTTGTCAGACAATATATTAAAGTATTTCCCACAAATCACAATGACTGTAAATCTTCTGGAGATGACAAAGACTATGTTGAATCTAGGGGTGAAACTTGCAGGCGGTAGCAGATATAATGTTGAAGACTCCCAATCTGTTGGAACTTATAAAAAAAGTGTGTCATATACAGTAAATATAGATTTTAAAAAGTGGAACGGGCAGATGAGACAAGAGATTACAGATCCTGTTAGATCGGAAGAGCACACGTCTGAACTCCAGTCACACTTGAATCTCGTATGCCGTCTTCTGCTTGAAAAAAAAAAAAAATAATCACACATAACATATAGATAAAGAAA

>NODE_51288 (Botourmiaviridae), RNA dependent RNA polymerase

TTTTTTTTTTTTTCAAGCAGAAGACGGCATACGAGATTACAAGGTGACTGGAGTTCAGACGTGTGCTCTTCCGATCTCTCCTCGTAAAATTCTACTAATTTCTTGGCACGACCGCGTAGCCGGCCGCTAGCAGTACAACCATCCTCGCACTCGTCGTGAAGCACCGCATACGGGTTAGCTTCGAGCGCTTGGTCATTACTGTTAGTAAGAGGTTCTAAGTAATCCTCTAAAACTTTGTCCGTCACAATTTCCTTATCCTCTTGCGGTCCGAGACGGGCTATTGCCTCGGTAATCACCCACTCCGGGTCATAAGTCGCCCTCTTCGAGAGCAACTCACCCCGGAACGTGTGGGCTCCTTTATAAGGAAAATTACGAAACGCAATAGGGAAAGGGGTAGCACGGGGAGGAGGAGCCGGTGTAGACTTTTGGGTGCAATAAAGCCTAGGATAAATACCGTTCTCCAACTGCCAATCTCGTTCCATCGGGCAACGACGATCACGGCAGCATATCACTGCCTCCCCCCAGATCGGAAGAGCGTCGTGTAGGGAAAGAGTGTAGATCTCGGTGGTCGCCGTATCATTAAAAAAAAAA

>NODE_53114 (Botourmiaviridae), RNA dependent RNA polymerase

TATGTTGTTTTTTTTCAAGCAGAAGACGGCATACGAGATGATCTGGTGACTGGAGTTCAGACGTGTGCTCTTCCGATCTGCCCGATGAAAACACTAGCTCGTACCGACATTCGCCGGAAAATTCTTCCACATTCCAATTACCACCCTCCTTCCTCCGGTAACGCCGGGTAGCATTTCCGTTTGGAATAAAAGGGGCACGCTGGCGGTCCCATCCCTTTTCTATGTTTTGCCTAAGGGCTCGTCGAAACTTACCCAAATGCTCCACATCGACAGCAACTGGTTGGAACCTAGCCTGTTTCCACTGGTCGAGCTTCTGTTCGAAACGAGGCAAACAGAACTTGCAACAAGATTTCTCAAGTTTTTGTATTGTCTTGAAGCTCAATTCATCAATAGGGCTAAGTCTATCAATGAAGCATTGTCGAACGGCACCGCGGAGATCTCCGCAGATCATGTGCTGGGGTACTTCTTTCGCTGAACGAGTCATACCAGATCGGAAGAGCGTCGTGTAGGGAAAGAGTGTAGATCTCGGTGGTCGCCGTATCATTAAAAAAAAAAAACTAAGACAAGTCGTGTGGAGGGTTG

>NODE_54016 (Mitoviridae), RdRP

GCCAACTTCATATCTTATGGAGTTACCATCCTTAGTATGGAAACTTATTGTCTCAAGGATTCTATTTCAAGATCATGCATATTGCATGTCTATCATTTCTGATAGTAATCTTGCTTGTAGTTTCCTTGGGAATCTATCCGTAGCAGAACTCAAATCCAATGATCAAAACTTATGTTCATTAAGTTCTCAGTTATGATGTGGATTTTGATTAAAAGTCCTATCAGTTTCCTTTAAGTTTTTAATAACCTTAAAGCACTGATCATGAAGTTTCTTTAGAGCTAATTGTGTATAGTAATCAACTATTGCAATTAATCTAAATTTACCTTCAGGATCTTTTATCACTTCAATTTTTCCCAAATCATTATGTTTTGGTTTAAATTTTGAAGAATTTTCCACAAAATAACTATAGGATTTTGAAATGAAATCAATACCGGTTTCACTAAGGACATTAAATAGTCTTTGTAAACTATAATAATTATAATTATTAAAATTTACTAAAGCACTATTTGATGCTTTACCTTGTGGCCCTCCCTTTTGTGATACATAAATATCACTGAGAGAGAACTGCATATTATCT

>NODE_57998 (unclassified Riboviria), putative nucleocapsid

GTTCAGACGTGTGCTCTTCCGATCTGGGGGCTATGGCTGCGTATGCCAGGCGCAGGGTTTTGCCGGTTGGTTCCTTTGGTAGGGTTGTGGCTGAGTCGGATGTGGTCGCTGAGAGGTCCTTTGTTAAGGGTTCGACCACCGTTGGTGATACTAGCGGTTTGGCTGGTGTTGCTGCGTTGGTGCTCCCGGTCTGTGGGGGTAAGACGTCGCTTGCTGGTGTTAGGCCGTGGCTTAAGGACGTTGATCAGTTGTGTGACCCCCGGGCTAATGAGGAGTTGTGTGCACTTCGGTCTGAGGCTAAGTTGACTGGGGACTGGGCTAGGTATAATGAGCTTTGGATGTCCTTTGTCAGGCCGAAGTTGGAGTTTGGTGATGTTGTTATGGTGCAGGATATTGAGTTGGCCACTGCGTTGGGTGTGGCTGATGCTAGGTGTGTTGTGCCGCCTTATGACGATTGGATTAAGGCGGTTGTGGCTAGGGGTGGAGTCGGGGGAGATCGGGAGAGCGTCGGGTTGGGGAAGAGTGTAGGTTTCGGGGGGTGCCGGATCCTTAAAAAAAA

>NODE_58741 (Mitoviridae), RNA-dependent RNA polymerase

AATCGGTGACGAGAGTTCATTATTTTTTTTTTTCAAGCAGAAGACGGCATACGAGATGTAGCCGTGACTGGAGTTCAGACGTGTGCTCTTCCGATCTTGACAGACGAAAATAATAGAAAATTATGACGATGAAAATCGAAATAATTTAATCTATTATCCAACTTTTGTTGGACTTTATAACTACATTAACAATATCAAAACTAGAACAAAAAGATGGAGTGGTTCTGAAGAAATTTCAGAACTAACACAAGATCTTAATGTTATAGATGTTGATAAAGTTTTCAGTAAGGAACGATCTAAATATGATCGGCTTCTTACTATAGGTAAGAGTTTAGAAGTAGGTTTCACTAACATCAATAAAACTGAAGAGATCTATTATGGATCTGCAACAGTTGAATCATCATTGACACCAAAAGGTATGCAATTATGATTCTCTAAATCTATTACAAAAGATGTAATGGACCAGATTATTGAAGGTAAGTGAGAACCTCCAAAACCTCAAATGTCATAGATCGGAAGAGCACACGTCTGAACTCCAGTCACTAGCTTATCTCGT

>NODE_59063 (Botourmiaviridae), RNA dependent RNA polymerase

GAAATCAGAGCGTTTGACGTCTCCTCTGACACACCAACCAAAAGAGGTTAGGTGGTCATACAGGGCCTCATGCACCGGACGTAGAATTCTCTTCACGTACGCTGGCTGCATGGTCACAACACGAAATTTTCCCTTTGTTTTGGCGACCCCTACCCTAAGGCAGGAGACATCTTCGCAAGGGTCGGAGCAGACGGCCAAAGTGCCGCCATTAGCTCTCCGATTCTCGAAGCAACCCTGTTGGTCGGGGACGTACCCACGCTCTTTATCCAAACCCCACTCGTTTCCCAACCAGAAATCTTTCCTCCGTGCCTCCAGTTTCCTCCCCCATCCTTCGGTGAGCTCGCGAACACGCCGCTTAAGGATCCAGAGCGGGTCAAAAGACCAATCACACTCCGGCATCGCCGCTGGTTGAGCAACGAAGTTTTGCCAAGCAGCGCGAGCCCGGGAGCCCGCAATAATATCGCAGGGAGGACAGCGACAATCAAAGATTCTCTTCACACTCTTTACAAGTGATCCAAATTTCTCTAATGTCTTAGATCGGAAGAGCACACGTCT

>NODE_60104 (Rhabdoviridae), L protein

TGTTTGTGAGAGTAGTAGTTTTTACTTTATGGTTTTTTTTTTTTCAAGCAGAAGACGGCATACGAGATTCAAGTGTGACTGGAGTTCAGACGTGTGCTCTTCCGATCTTGGGGGAAAGACTGAATTGTCTCATTTATGCGATGATTTCTGAAAGTTACAAATATGATGTGGCACCTGTATCATCAGACAAACTACTAGAAGTTATATTATGGGGGGATGATATATTAAGATTCCATGACTCTGAGGCATTCCTATTCTTAAGCAATTATGAAGCTTTGATAGTGGGAGAAATATTAAGTAGAGGTGATCAAGATCTTTGGTATGTGGAAAGATTCAGGACCTCCTTGCATGAAGACATAATTCAGGAGACGCCCTGGGCAAAGAAGCATTATGAAAGATTGGAGAGTATAATATCTGATATGAGCGTAGATCAGTTATCTGATATTCATGGAATCTACAGGATATGGGGACAGATCGGATGAGCGTCGTGTAGGGAAAGAGTGTAGATCTCGGTGGTCGCCGTATCATTAAAAAAAAAAAATTTAAGCCA

>NODE_60865 (Caulimoviridae), Enzymatic polyprotein

GATGATAAACCTAAAGATGAAAAAGAATTACAGAGATTTTTAGGAATTTTAAATTATATAGGAGATTTTATTCCTTACTTATCTGAATTAAGAAAACCTTTTCAAAAATTGTTAAAGAAAAATCAGACTTATACTTGGGGTCCAGAACAAGATAAAAATATAGAGTTAATAAAAAAGATTTGTAGAAATTTACCTAAATTGGAATTACCTAAACAAACAGATGAAATTATAGTTGAAACAGATGCTTGTGAAGAAAGTTGGGGTGGAGTCTTAAAAGTTAAAAAAGATGATAAAGAAGAAATATGTAGATATACTAGTGGAACATTTACAATTTCTGAGAAAAATTATCATATAAATGAAAAAGAATTACTTGCTGTAGTTCGTAGTTTTTCAAAATTTAGTTATTTTATACAACCTAAGAAATTTCTATTAAGAACTGATAATACACAGGTTAAATCTTTTATTAGAAATAACTTACCTTCTAAACCAGAATACAAGAGAATAATCAGATGGCAAATGCTTTTGCAAGAATATGATTTTGAAATTG

>NODE_68365 (Botourmiaviridae), RNA dependent RNA polymerase

CCTCCTCTCAGATACACACTCCTAGTTATTTTTTTTTTTAATGATACGGCGCCCACCCAGATCTACACTCTTTCCCTACACGACGCTCTTCCGATCTCCCTGACACGCTCAATCTCTTCTTTCATTGCCTCATGTTCTTCATCCCTAGTTAGGGAGTAGTTTTCAGGCCGAGGTGCCATACTAATTACGCCCTGTTCGGTCGGTTTCACACGATCAGGCAAGCTGGTGATAGCCTTTCTAATAACTTTGTCTTGTCGGCAGATGGCGACTAGCGGATAGGGGATCTCTGACAAATGCTTGTCAGCCTGTTTGGCCAACGTCCTCGCATTACGCCGTACTATCTTCCTAAAGGTCTTCCCATCTGGGCTGGCCTGGGCTGCAAAACCCAAGACATCCTCTACACCAGCATCCATCCACATCGACGCTGCGTTAAACTTCCTTTGCTTACAGCCCCAGATCGGAGGAGCGTCGTGTAGGGAAAGAGTGTAGATCTCGGTGGTCGCCGTATCATTAAAAAAAAA

>NODE_74445 (Caulimoviridae), putative coat protein

AGAAATGCTTTCACATACAATAATGACTTATCAGATAATGATAAAAAAGAAGTCATAGTAAGATCATTAACAGGTATGGCAAAAGAATATTATGATAATCTAAGCCAAAATGTAAGAGATGGATTGTTTGCTGGAACACATACAGATTGGTTAGGAAGAATAAGACAATTATTTTTGGTAGAATTTAATGGAACAACTGATCCATCTGAAGAATCTATTGATTTTAAAATATTATTACAAAGAATTATTCTTTGTGATATATGTTATATTGATGAATATATATGTTTATTTCAAAAATATTATTATAAGGCTAAAATAACTACTGAAGAAAAACCAACATATTTAGATTTATTCTTTTCTAAAATACCAGGAAAGTATGGAGAAAAATTTATAAAAGATTATCAACCAGGAACAATAGATTCTTTGGGAAAAAGAATAAAACATGTTCAACAATGCATATCTTATTATTGTAATGAATTAAGCCTATATAAAAAGATGGCCAG
