# Supplementary material for: Expanding the environmental virome: Infection profile in a native rainforest tree species
Source: Front Microbiol. 2022 Aug 4;13:874319. doi: 10.3389/fmicb.2022.874319 (PMC9387356; doi:10.3389/fmicb.2022.874319)
Supplement: Supplementary file 2 [file Data_Sheet_2.pdf]

Supplementary Material

Expanding the environmental virome: Infection profile in a native rainforest tree species.

Anderson Carvalho Vieira[1,#], Ícaro Santos Lopes[2,#], Paula Luize Camargos Fonseca[3], Roenick Proveti Olmo[4], Letícia Maróstica de Vasconcelos[1], Carlos Priminho Pirovani[3], Fernanda Amato Gaiotto[1,*], Eric Roberto Guimarães Rocha Aguiar[1,*]

[1]Department of Biological Science (DCB), Center of Biotechnology and Genetics (CBG), Universidade Estadual de Santa Cruz (UESC), Ilhéus, Bahia, CEP 45662-900, Brazil
[2]Department of Biochemistry and Immunology, Instituto de Ciências Biológicas, Universidade Federal de Minas Gerais, Belo Horizonte, Minas Gerais, CEP 30270-901, Brazil;
[3]Department of Genetics, Instituto de Ciências Biológicas, Universidade Federal de Minas Gerais, Belo Horizonte, Minas Gerais, CEP 30270-901, Brazil
[4]Université de Strasbourg, CNRS UPR9022, Inserm, Strasbourg, France

[#]These authors contributed equally to his work.
[*]Both corresponding authors contributed equally to this work.

Figure S1. Metagenomics analysis based on assembled transcripts. Analysis was performed using Kaiju webserver with NCBI NR database.

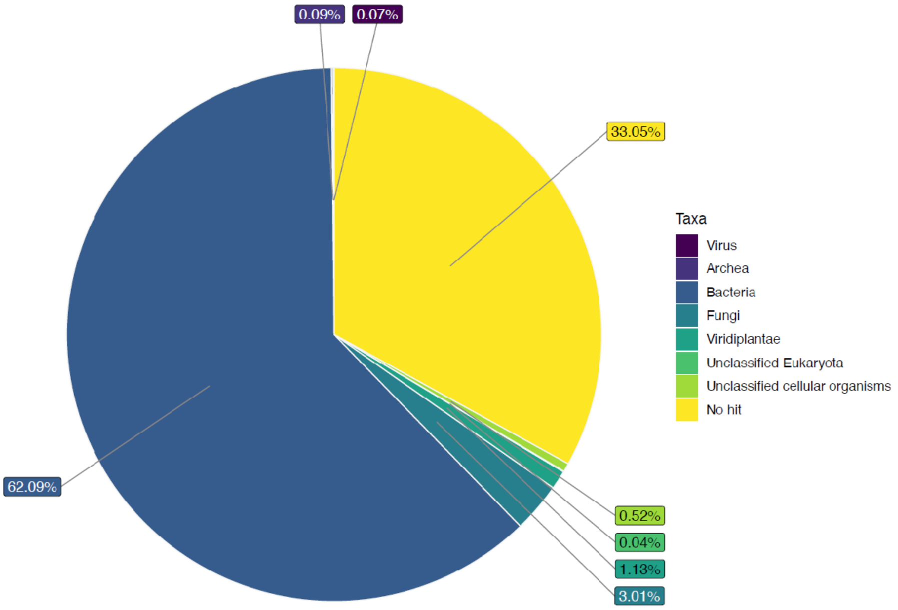

Figure S2. Structural and domain annotation of *Skokie picorna-like virus*.

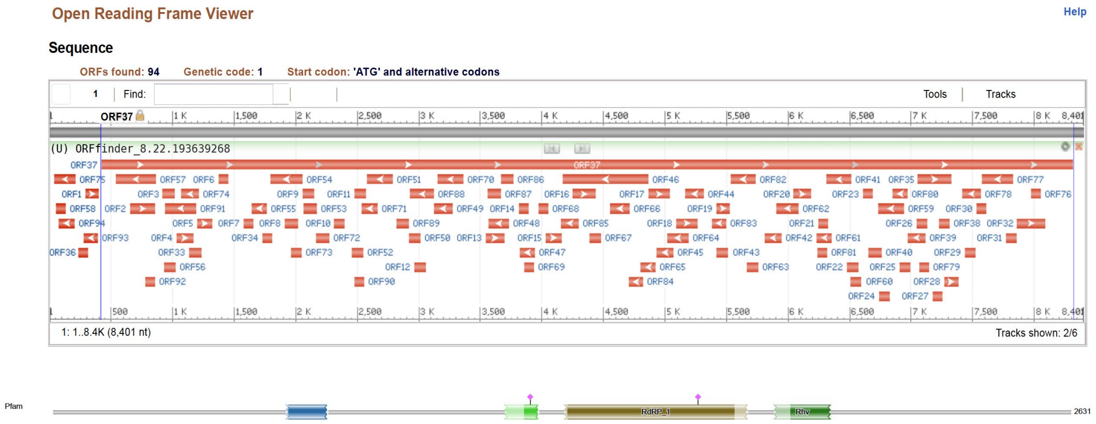

Figure S3. Structural and domain annotation of closest sequences from *Bromoviridae* family.

Lilac ring mottle virus – Movement protein

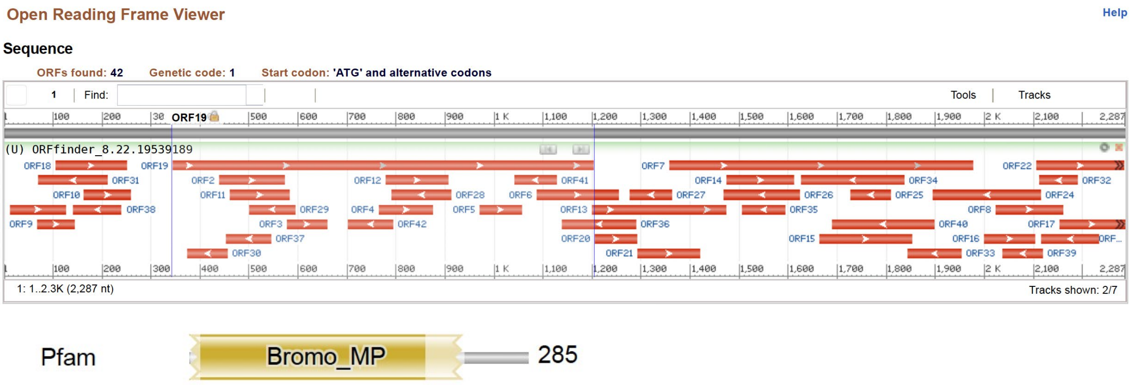

Citrus leaf rugose virus- RdRP

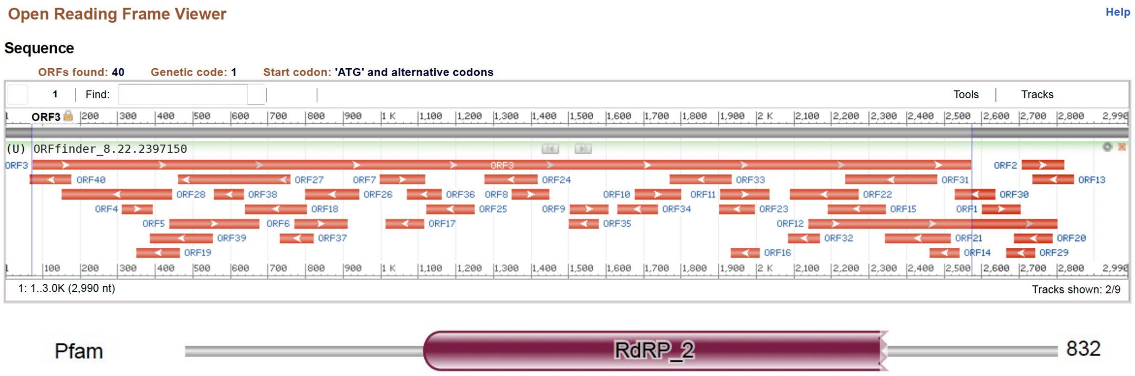

Tomato necrotic streak virus - Replicase

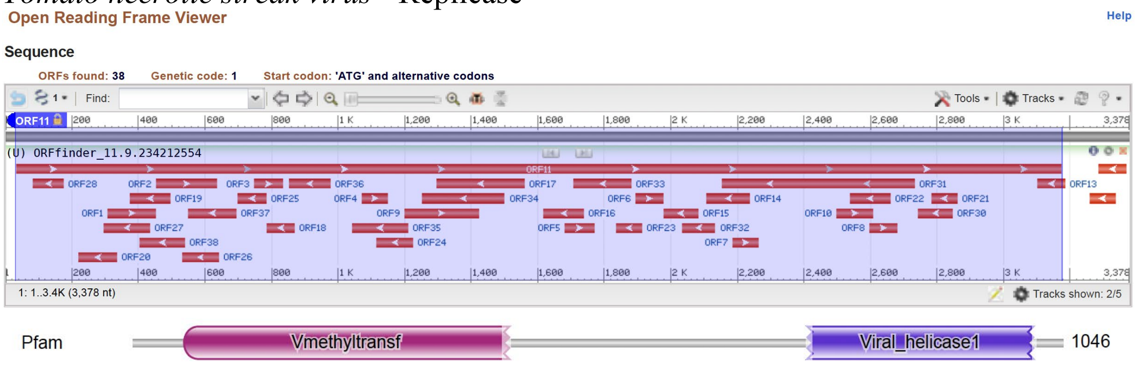

Figure S4. Structural and domain annotation of C*hicken genomovirus mg4_1247*.

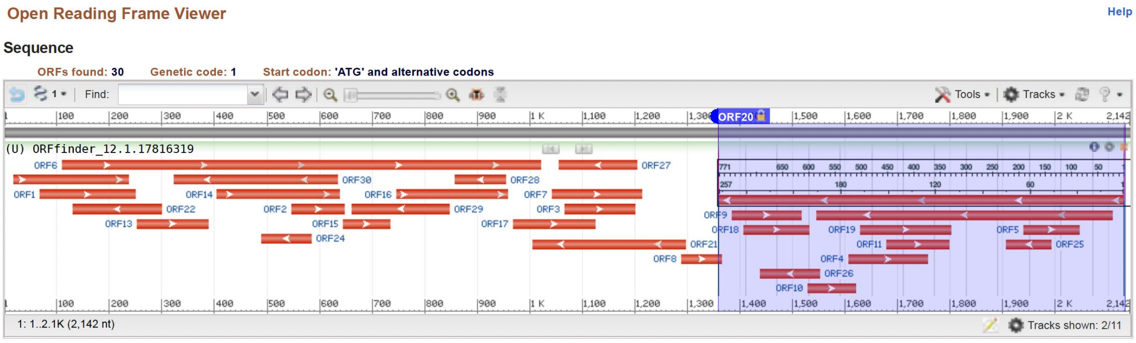

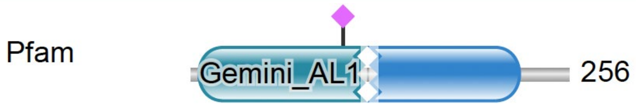

Table S1. Field collection and overview of samples and RNA deep sequencing of *Carpotroche brasiliensis* organs .

Sample	Ecosystems	WGS 84 (World Geodetic System)	ID	Organ	Sex	Reads after sequencing	Reads after trimmomatic
1	Agroforestry	14°05'15"S, 39°14'11"W	CVB 07	flower bud	♀	1.843.178	1.093.210 (70.02%)
2	Agroforestry	14°05'17"S, 39°14'19"W	CVB 14	flower bud	♂	1.092.821	772.653 (70.70%)
5	Agroforestry	14°05'09"S, 39°14'19"W	CVB 12	flower bud	♂	1.726.172	1.231.572 (71.35%)
25	Agroforestry	14°05'15"S, 39°14'11"W	CVB 07	flower bud	♀	1.736.441	1.247.547 (71.85%)
26	Agroforestry	14°05'15"S, 39°14'11"W	CVB 08	flower bud	♀	1.685.607	1.135.844 (67.38%)
29	Agroforestry	14°05'17"S, 39°14'19"W	CVB 14	flower	♂	1.870.044	1.061.472 (71.34%)
30	Agroforestry	14°05'12"S, 39°14'09"W	CVB 04	fruit		1.487.803	1.076.762 (69.45%)
50	Agroforestry	14°05'15"S, 39°14'11"W	CVB 08	seed	♀	1.913.780	1.689.561 (67.88%)
65	Agroforestry	14°05'12"S, 39°14'09"W	CVB 10	leaf	♀	2.488.977	1.333.571 (70.60%)
74	Agroforestry	14°05'15"S, 39°14'09"W	CVB 11	root	---	1.923.348	1.225.955 (65.64%)
81	Agroforestry	14°05'15"S, 39°14'11"W	CVB 09	seed	♀	1.464.053	1.288.500 (69.91%)
6	Forest	14°06'51"S, 39°16'15"W	A 03	flower bud	♀	1.710.431	1.200.003 (70.16%)
10	Forest	14°06'54"S, 39°16'19"W	A 04	flower bud	♀	1.667.608	1.159.280 (69.52%)
67	Forest	14°06'61"S, 39°16'25"W	A 10	flower bud	♀	1.664.347	1.217.672 (73.16%)
17	Forest	14°06'51"S, 39°16'15"W	A 06	flower bud	♂	1.965.345	1.376.394 (70.03%)
7	Forest	14°06'54"S, 39°16'19"W	A 07	flower	♀	2.315.336	1.631.935 (70.48%)
46	Forest	14°06'56"S, 39°16'18"W	A 15	seed	♀	1.538.784	1.369.276 (71.55%)
72	Forest	14°06'56"S, 39°16'18"W	A 16	leaf	♂	1.888.874	1.311.732 (68.20%)
21	Forest	14°06'56"S, 39°16'18"W	A 17	root	---	1.870.044	1.335.482 (71.41%)
78	Forest	14°06'56"S, 39°16'18"W	A 15	seed	♀	1.867.660	930.949 (63.59%)

Proteomics data

	Ecosystems	WGS 84 (World Geodetic System)	Number of samples	Organ	Sex	Total peptides produced	Peptides matching viral sequences
	Agroforestry	14°05'09.6"S 39°14'09.6"W	2 X (3 replicates)	seed	♀	26977	201

Table S2. Overview of the transcriptome assembly.

Metric	Amount
Number of contigs	281,643
Number of bases in all contigs (bp)	160,909,318
N50	481
Longest Contig (bp)	56,208
Median contig size (bp)	423

Table S4. Overview of sequences showing similarity to viral species.

Contigs	Length	Organism hit	Protein hit	Alignment method	e-value	Cover
Carpotroche-associated picornavirus	8636	Skokie picorna-like virus	polyprotein	BlastN	0.0	96%
Carpotroche-associated ilarvirus	2442	Lilac ring mottle virus	movement protein	BlastN	0.0	89%
Carpotroche-associated ilarvirus	2954	Citrus leaf rugose virus	replicase protein 2a	BlastN	0.0	65%
Carpotroche-associated ilarvirus	3467	Spinach latent virus	replicase	BlastN	1E-127	62%
Carpotroche-associated genomovirus	943	Chicken genomovirus mg4_1247	replication-associated protein	BlastX	2E-050	62%
NODE_9936	1673	Biomphalaria virus 2	polyprotein	BlastN	3E-056	35%
NODE_15442	1321	Maize fine streak nucleorhabdovirus	N	BlastX	7E-102	88%
NODE_18845	1156	Maize fine streak nucleorhabdovirus	G	BlastX	9E-037	66%
NODE_23163	1005	Beta vulgaris mitovirus 1	RNA-dependent RNA polymerase	BlastX	4E-021	26%
NODE_23836	983	Maize fine streak nucleorhabdovirus	L	BlastX	3E-076	72%
NODE_33142	769	Erysiphe necator associated ourmia-like virus 41	RNA-dependent RNA polymerase	BlastN	1E-171	70%
NODE_39152	686	Maize fine streak nucleorhabdovirus	N	BlastX	6E-026	89%
NODE_39399	683	Botrytis cinerea mitovirus 8	RNA-dependent RNA polymerase	BlastN	2E-021	17%
NODE_41237	664	Maize fine streak nucleorhabdovirus	L	BlastX	4E-091	86%
NODE_41497	662	Plasmopara viticola lesion associated ourmia-like virus 87	RNA-dependent RNA polymerase	BlastN	0.0	87%
NODE_45025	632	Hubei narna-like virus 2	RNA-dependent RNA polymerase	BlastX	4E-030	70%
NODE_47371	615	Macrophomina phaseolina mitovirus 3	putative replicase	BlastN	8E-033	50%
NODE_48513	607	Maize fine streak nucleorhabdovirus	N	BlastX	4E-018	41%
NODE_48606	607	Plasmopara viticola lesion associated ourmia-like virus 41	RNA-dependent RNA polymerase	BlastN	9E-178	70%
NODE_50646	594	Maize fine streak nucleorhabdovirus	L	BlastX	6E-051	74%
NODE_51288	591	Erysiphales ourmia-like virus 2	RNA-dependent RNA polymerase	BlastN	0.0	75%
NODE_53114	582	Erysiphales ourmia-like virus 2	RNA-dependent RNA polymerase	BlastN	0.0	70%
NODE_54016	577	Fusarium andiyazi mitovirus 1	RdRP	BlastN	0.0	100%
NODE_57998	559	Plasmopara viticola lesion associated mononegaambi virus 2	putative nucleocapsid	BlastX	8E-09	58%
NODE_58741	556	Fusarium circinatum mitovirus 2-1	RNA-dependent RNA polymerase	BlastN	5E-180	74%
NODE_59063	555	Plasmopara viticola lesion associated ourmia-like virus 59	RNA-dependent RNA polymerase	BlastN	2E-127	93%
NODE_60104	550	Maize fine streak nucleorhabdovirus	L	BlastX	6E-024	66%
NODE_60865	547	Carnation etched ring virus	Enzymatic polyprotein	BlastX	4E-053	99%

NODE_68365	521	Plasmopara viticola lesion associated ourmia-like virus 90	RNA-dependent RNA polymerase	BlastN	5E-142	68%
NODE_74445	503	Tobacco vein clearing virus	putative coat protein	BlastX	2E-015	80%
